# Structural Determinants of Misfolding in Multidomain Proteins

**DOI:** 10.1371/journal.pcbi.1004933

**Published:** 2016-05-10

**Authors:** Pengfei Tian, Robert B. Best

**Affiliations:** Laboratory of Chemical Physics, National Institute of Diabetes and Digestive and Kidney Diseases, National Institutes of Health, Bethesda, Maryland, United States of America; Centre National de la Recherche Scientifique, FRANCE

## Abstract

Recent single molecule experiments, using either atomic force microscopy (AFM) or Förster resonance energy transfer (FRET) have shown that multidomain proteins containing tandem repeats may form stable misfolded structures. Topology-based simulation models have been used successfully to generate models for these structures with domain-swapped features, fully consistent with the available data. However, it is also known that some multidomain protein folds exhibit no evidence for misfolding, even when adjacent domains have identical sequences. Here we pose the question: what factors influence the propensity of a given fold to undergo domain-swapped misfolding? Using a coarse-grained simulation model, we can reproduce the known propensities of multidomain proteins to form domain-swapped misfolds, where data is available. Contrary to what might be naively expected based on the previously described misfolding mechanism, we find that the extent of misfolding is not determined by the relative folding rates or barrier heights for forming the domains present in the initial intermediates leading to folded or misfolded structures. Instead, it appears that the propensity is more closely related to the relative stability of the domains present in folded and misfolded intermediates. We show that these findings can be rationalized if the folded and misfolded domains are part of the same folding funnel, with commitment to one structure or the other occurring only at a relatively late stage of folding. Nonetheless, the results are still fully consistent with the kinetic models previously proposed to explain misfolding, with a specific interpretation of the observed rate coefficients. Finally, we investigate the relation between interdomain linker length and misfolding, and propose a simple alchemical model to predict the propensity for domain-swapped misfolding of multidomain proteins.

## Author Summary

Multidomain proteins with tandem repeats are abundant in eukaryotic proteins. Recent studies have shown that such domains may have a propensity for forming domain-swapped misfolded species which are stable for long periods, and therefore a potential hazard in the cell. However, for some types of tandem domains, no detectable misfolding was observed. In this work, we use coarse-grained structure-based folding models to address two central questions regarding misfolding of multidomain proteins. First, what are the possible structural topologies of the misfolds for a given domain, and what determines their relative abundance? Second, what is the effect of the topology of the domains on their propensity for misfolding? We show how the propensity of a given domain to misfold can be correlated with the *stability* of domains present in the intermediates on the folding and misfolding pathways, consistent with the energy landscape view of protein folding. Based on these observations, we propose a simplified model that can be used to predict misfolding propensity for other multidomain proteins.

## Introduction

**Fig 1:**
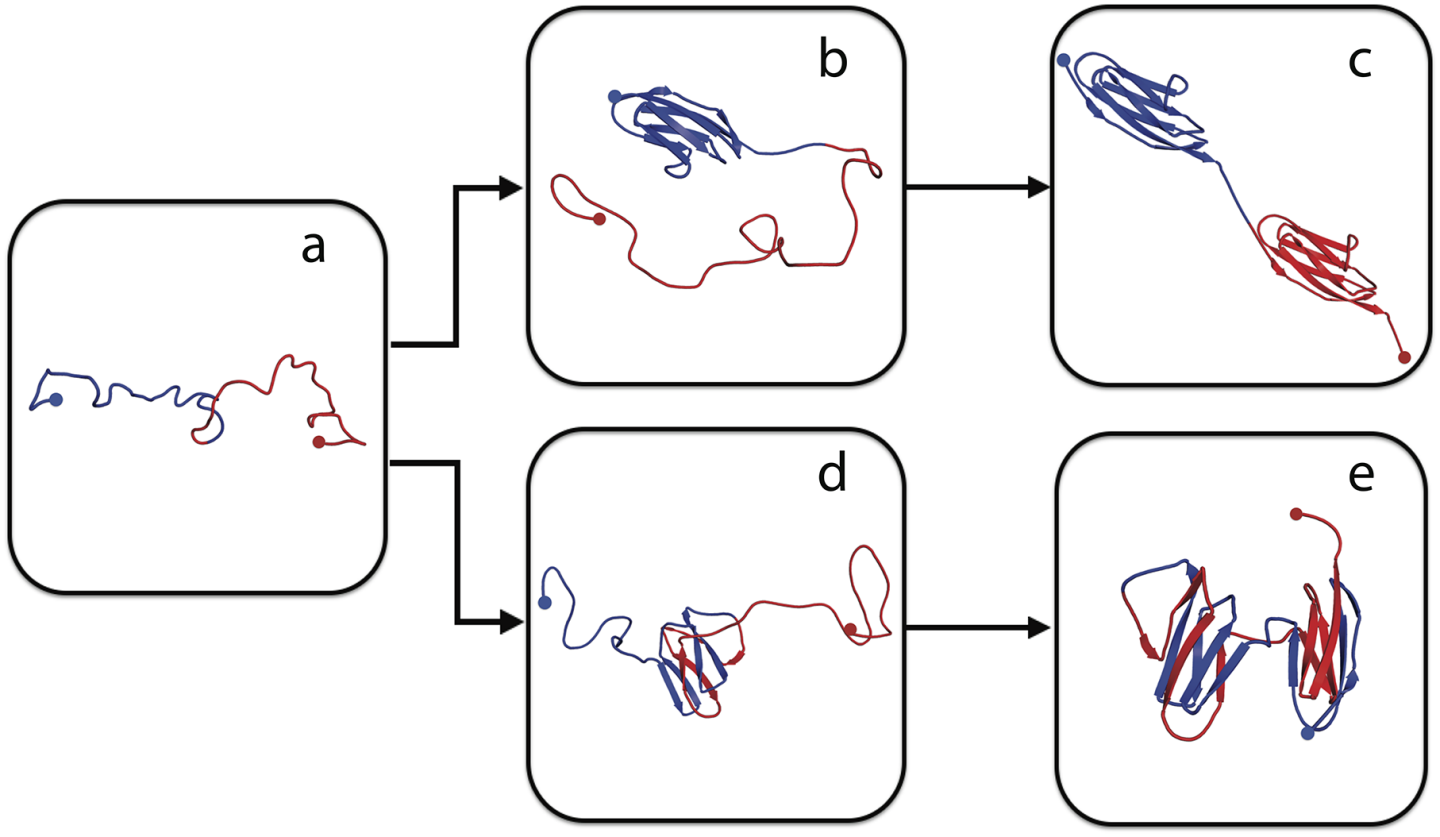
Misfolding mechanism of tandem domains. The schematic shows the native-like stable intermediates populated *en route* to native folding (upper) or misfolding (lower), and used to explain single-molecule and ensemble folding kinetics [12]. The correctly folded dimer (c) is formed from the unfolded chain (a) via an intermediate (b) in which either of the domains folds natively. The misfolded dimers (e) form via initial formation of a domain-swapped “central domain” (d) formed by the central regions of the sequence, followed by a “terminal domain” formed by the terminal regions of the sequence. The blue and red dots indicate the N- and C- terminal respectively, in each case. The N- and C-terminal halves of the chain are also coloured in blue and red respectively.

Protein misfolding and aggregation are well-known for their association with amyloidosis and other diseases [1, 2]. Proteins with two or more domains are abundant in higher organisms, accounting for up to 70% of all eukaryotic proteins, and domain-repeat proteins in particular occupy a fraction up to 20% of the proteomes in multicellular organisms [3, 4], therefore their folding is of considerable relevance [5]. Since there is often some sequence similarity between domains with the same structure, it is easily possible to imagine that multidomain proteins containing repeats of domains with the same fold might be susceptible to misfolding. Indeed, misfolding of multidomain proteins has been observed in many protein families [6]. Single molecule techniques have been particularly powerful for studying folding/misfolding of such proteins, in particular Förster resonance energy transfer (FRET) and atomic force microscopy (AFM). For instance, recent studies using single-molecule FRET, in conjunction with coarse-grained simulations, have revealed the presence of domain-swapped misfolded states in tandem repeats of the immunoglobulin-like domain I27 from the muscle protein Titin [7] (an example is shown in Fig 1e). Domain-swapping [2] involves the exchange of secondary structure elements between two protein domains with the same structure. Remarkably, these misfolded states are stable for days, much longer than the unfolding time of a single Titin domain. The domain-swapped misfolds identified in the Titin I27 domains are also consistent with earlier observations of misfolding in the same protein by AFM, although not given a structural interpretation at the time [8]. In addition, AFM experiments have revealed what appears to be a similar type of misfolding in polyproteins consisting of eight tandem repeats of the same fibronectin type III domain from tenascin (TNfn3) [9], as well as in native constructs of tenascin [8], and between the N-terminal domains of human *γ*D-crystallin when linked in a synthetic oligomer [10].

In addition to domain-swapped misfolding, an alternative type of misfolded state is conceivable for polyproteins in which the sequences of adjacent domains are similar, namely the formation of amyloid-like species with parallel *β*-sheets. Theoretical work in fact made the prediction that such species would be formed in tandem repeats of titin domains [11]. Recently, time-resolved single-molecule FRET experiments on tandem domains of I27 have revealed a surprising number of intermediates formed at short times, which include an unexpected species that appears to be consistent with the previously suggested amyloid-like state [12]. However, since only the domain-swapped species persisted till long times, and therefore are the most likely to be problematic in cells, we focus on their formation in this work.

A simplified illustration of the mechanism for folding and misfolding, based on both coarse-grained simulations as well as single-molecule and ensemble kinetics [7, 12], is shown in Fig 1, using the Titin I27 domain as an example. Starting from the completely unfolded state in Fig 1a, correct folding would proceed via an intermediate in which either one of the domains is folded (Fig 1b), and finally to the fully folded state, Fig 1c. The domain-swapped misfolded state, an example of which is shown in Fig 1e, consists of two native-like folds which are in fact assembled by swapping of sequence elements from the N- and C-terminal portions of the protein. The final structure in Fig 1e comprises what we shall refer to as a “central domain” formed by the central regions of the sequence (on the left in Fig 1e) and a “terminal domain” formed from the N- and C-termini (on the right). The intermediate structure in Fig 1d, suggested by coarse-grained simulations [7], and supported by experiment [12], has only the central domain folded. This central domain can itself be viewed as a circular permutant [13] of the original native Titin I27 structure, as discussed further below.

While domain-swapped misfolding of tandem repeats has been identified in a number of proteins to date, there are several other proteins for which it does not occur to a detectable level. For instance, extensive sampling of repeated unfolding and folding of a polyprotein of Protein G (GB1) by AFM revealed no indication of misfolded states, in contrast to Titin [14]. Similarly, early AFM studies on polyUbiquitin also did not suggest misfolded intermediates in constant force unfolding [15–20], and lock-in AFM studies of refolding [21] were fully consistent with a two-state folding model, without misfolding. More recent AFM [22] studies have suggested the formation of partially folded or misfolded species, which have been attributed to partial domain swapping in simulations [23], but these are qualitatively different from the fully domain-swapped species considered here. Therefore, it is interesting to ask the general questions: when included in tandem repeats, what types of protein structures are most likely to form domain-swapped misfolded states, and by what mechanism?

**Fig 2:**
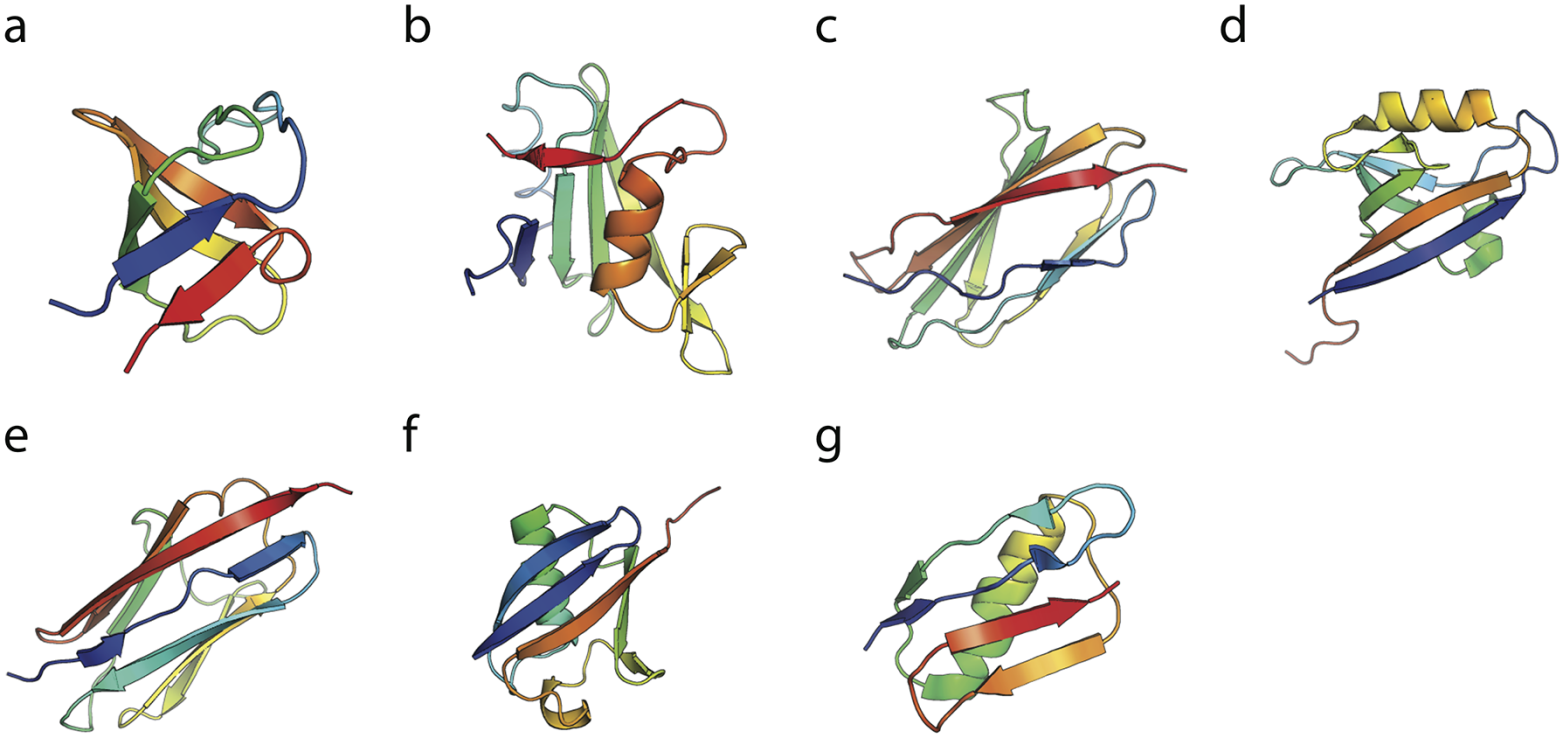
Native states of the single domains. The experimentally determined structure of a single domain of each of the protein domains studied here: (a) SH3, (b) SH2, (c) TNfn3, (d) PDZ, (e) Titin I27, (f) Ubiquitin and (g) Protein G. The PDB accession code are 1SHG, 1TZE, 1TEN, 2VWR, 1TIT, 1UBQ and 1GB1, respectively.

In order to investigate the misfolding propensity of different types of domains, we have chosen seven domains, based on (i) the superfamilies with the largest abundance of repeats in the human genome [24], (ii) proteins for which some experimental evidence for misfolding (or lack thereof) is available and (iii) proteins for which data on folding kinetics and stability is available for their circular permutants (only some of the proteins meet criterion (iii)). The circular permutant data are relevant because the misfolding intermediates suggested by simulations and experiment [7, 12] can be viewed as circular permutants of the original structure (Fig 1d). Each of the chosen proteins is illustrated in Fig 2 and described briefly in Materials and Methods. We study the folding and misfolding of the seven protein domains, using the same structure-based model as that successfully employed to treat Titin I27 [7, 12]. Molecular simulations are carried out to characterize the possible structural topologies of the misfolded intermediates and the mechanism of their formation. Our model is consistent with available experimental information for the systems studied, in terms of which proteins misfold and what misfolded structures they tend to form. We then investigated what factors influence the propensity of multidomain proteins to misfold. The simplest rationalization of the propensity of a multidomain protein for domain-swapped misfolding would seem to be offered by parameterizing a kinetic model based on the scheme shown in Fig 1, particularly for the steps Fig 1a–1b versus 1a–1d. We hypothesized that the propensity to misfold might be characterized in terms of the folding kinetics of the isolated circular permutants representing the domain-swapped intermediates in Fig 1d. However, contrary to this expectation, we found that the stability of such isolated domains, rather than their folding rate, is the main determinant of misfolding propensity. Although superficially this appears to differ from previously suggested kinetic models [12], it is completely consistent, with a specific interpretation of the rates. Building on this understanding, we developed a very simplified model which can be used to predict which domains are likely to be susceptible to domain-swapped misfolding. Finally, we have investigated the effect of the composition and length of the linker between the tandem repeats on the misfolding propensity.

## Materials and Methods

### Choice of proteins

Tandem Src homology 3 (SH3) domains (Fig 2a) are widely found in signal transduction proteins and they share functions such as mediating protein-protein interactions and regulating ligand binding [25]. Kinetic and thermodynamic properties of native and all the possible circular permutations of SH3 single domain have been well characterized [26]. Two different circular permutant constructs of the sequence are known to fold to a circularly permuted native conformation (PDB accession codes are 1TUC and 1TUD) that is similar to the wild-tpe (WT) protein [26].

With a similar function to the SH3 domains, Src homology 2 (SH2) domains (Fig 2b) are also involved in the mediation of intra- and intermolecular interactions that are important in signal transduction [27]. The SH2 domains are well-known from crystallographic analysis to form metastable domain-swapped dimers [28, 29].

Fibronectin type III (fn3) domains (Fig 2c) are highly abundant in multidomain proteins, and often involved in cell adhesion. We have chosen to study the third fn3 domain of human tenascin (TNfn3), which has been used as a model system to study the mechanical properties of this family. Single-molecule AFM experiments revealed that a small fraction (∼ 4%) of domains in native tenascin (i.e. the full tenascin protein containing both TNfn3 and other fn3 domains) [8], with a similar signature to that observed for I27. Subsequently, misfolding events have been identified in a polyprotein consisting of repeats of TNfn3 only [9]. Interestingly, a structure has been determined for a domain-swapped dimer of TNfn3 involving a small change of the loop between the second and third strand [30].

PDZ domains (Fig 2d) are one of the most common modular protein-interaction domains [31], recognizing specific-sequence motifs that occur at the C-terminus of target proteins or internal motifs that mimic the C-terminus structurally [32]. Naturally occurring circularly permuted PDZ domains have been well studied [33–35], and domain-swapped dimers of PDZ domains have been characterized by NMR spectroscopy [36, 37].

Titin (Fig 2e) is a giant protein spanning the entire muscle sarcomere [38]. The majority of titin’s I-band region functions as a molecular spring which maintains the structural arrangement and extensibility of muscle filaments [39]. The misfolding and aggregation properties of selected tandem Ig-like domains from the I-band of human Titin (I27, I28 and I32) have been extensively studied by FRET experiments [7, 24]. In the earlier work on tandem repeats of I27 domains, around 2% misfolding events were reported in repeated stretch-release cycles in AFM experiments [8]. A slightly larger fraction (∼ 6%) of misfolded species was identified in single-molecule FRET experiments and rationalized in terms of domain swapped intermediates, captured by coarse-grained simulations [7, 11].

In contrast, with the above misfolding-prone systems, there are certain polyprotein chains have been shown be resistant to misfolding, according to pulling experiments. For instance little evidence for misfolding was identified in a polyprotein of GB1 [14] (Fig 2g), with more than 99.8% of the chains (GB1)_8_ folding correctly in repetitive stretching–relaxation cycles [14].

Lastly, we consider polyUbiquitin (Fig 2f), for which there is conflicting experimental evidence on misfolding. Initial force microscopy studies showed only the formation of native folds [15], with no misfolding. Later work suggested the formation of collapsed intermediates [22], however the signature change in molecular extension of these was different from that expected for fully domain-swapped misfolds. A separate study using a lock-in AFM [21] found Ubiquitin to conform closely to expectations for a two-state folder, without evidence of misfolding. For this protein, there is a strong imperative to avoid misfolding, since Ubiquitin is initially expressed as a tandem polyUbiquitin chain in which adjacent domains have 100% sequence identity, yet this molecule is critical for maintaining cellular homeostasis [40].

### Coarse grained simulation model

A coarse grained structure-based (Go-like) model similar to the earlier work is employed for the study here [7, 41]. Each residue is represented by one bead, native interactions are attractive and the relative contact energies are set according to the Miyazawa–Jernigan matrix. The model is based on that described by Karanicolas and Brooks [41], but with native-like interactions allowed to occur between domains as well as within the same domain, as described below [7]. All the simulations are run under a modified version of GROMACS [42]. For the seven species we studied in this work, the native structures of single domains that were used to construct the models for SH3, SH2, PDZ, TNfn3, Titin I27, GB1 and Ubiquitin correspond to PDB entries 1SHG [43], 1TZE [44], 2VWR, 1TEN [45], 1TIT [46], 1GB1 [47] and 1UBQ [48] respectively. For the single domains of SH3(1SHG), TNfn3(1TEN) and GB1(1GB1), additional linker sequences of Asp-Glu-Thr-Gly, Gly-Leu and Arg-Ser, respectively, are added between the two domains to mimic the constructs used in the corresponding experiments [9, 14, 26]. Construction of the Titin I27 model was described in our previous work [7].

In order to allow for domain-swapped misfolding, the native contact potentials within a single domain are also allowed to occur between corresponding residues in different domains, with equal strength. Specifically, considering each single repeat of the dimeric tandem that has *L* amino acids, given any pair of residues (with indices *i* and *j*) that are the native interactions within a single domain, the interaction energy for the intradomain interaction (*E*_*i*,*j*_(*r*)) is the same as the interdomain interaction between the residue (*i* or *j*) and the corresponding residue (*j* + *L* or *i* + *L*) in the adjacent domain, i.e. *E*_*i*,*j*_(*r*) = *E*_*i*+*L*,*j*_(*r*) = *E*_*i*,*j*+*L*_(*r*) = *E*_*i*+*L*,*j*+*L*_(*r*).

### Kinetic folding simulation of dimeric tandem

To investigate the folding kinetics of the dimeric tandem, a total of 1024 independent simulations are performed on each system for a duration of 12 microseconds each. Different misfolding propensities are observed at the end of the simulations. With the exception of Ubiquitin and GB1, the vast majority of the simulations reached stable native states with separately folded domains. A small fraction of simulations form stable domain-swapped misfolded states. All the simulations are started from a fully extended structure, and run using Langevin dynamics with a friction of 0.1 ps^−1^ and a time step of 10 fs.

### Folding reaction coordinates

We note that all the generated domain-swapped misfolding structures, containing the central and terminal domains, can be monitored by a reaction coordinate based on circular permutated native-like contact sets. Each circularly permuted misfold can be characterized according to the loop position *K* in sequence where the native domain would be cut to form the circular permutant (*K* = 0 corresponds to the native fold). If a native contact *C*_native_ = (*i*,*j*) exists between residues *i* and *j* in the native fold, the corresponding native-like contacts for the central (*C*_in_(*K*)) and terminal domains (*C*_out_(*K*)) of the domain swapped conformation are generated as

$$\begin{array}{ll} C_{in}(K) & =(i+\Theta (K-i)L,j+\Theta (K-j)L),\\ C_{out}(K) & =(i+\Theta (i-K)L,j+\Theta (j-K)L), \end{array}$$

where Θ(*x*) is the Heaviside step function and *L* is the length of each single domain (plus interdomain linker). *S*_in,*K*_ is the set of native-like contacts *C*_in_ of the central domain, and *S*_out,*K*_ is the set of all the native-like contacts *C*_out_ of the terminal domain. *S*_in,*K*_ and *S*_out,*K*_ can be used to define a contact-based reaction coordinate to analyze the kinetics of the dimeric tandem misfolding. The corresponding fraction of contacts for the central domain could be calculated by:

$$Q_{K}\left(\chi \right)=\frac{1}{N}\underset{\left(i,j\right)\in S_{\text{in},K}}{\sum }\frac{1}{1+e^{\beta \left(r_{ij}\left(\chi \right)-\lambda r_{ij}^{0}\right)}},$$ (1)

where *N* is the total number of domain swapped contacts, *S*_*K*_ = *S*_in,*K*_ ∪ *S*_out,*K*_ (equal to the total number of native contacts), *r*_*ij*_(*χ*) is the distance between residue *i* and *j* in the protein configuration *χ*. $r_{ij}^{0}$ is the corresponding distance in the native structure for native-like contacts, *β* = 50 *nm*^−1^ and *λ* = 1.2 is used to account for fluctuations about the native contact distance.

### Equilibrium properties and free energy surfaces

The equilibrium properties of a single domain of each system are obtained from umbrella sampling along the native contacts *Q* as the reaction coordinate. The obtained melting temperature of each system is listed in Table A in S1 Text. A temperature at which the folding barrier Δ*G*_*f*_ of approximately ∼ 2.5 *k*_*B*_*T* is chosen for the 2-domain tandem simulations for reasons described below. The stability Δ*G*_*s*_ is calculated as

$$\Delta G_{s}=-k_{B}T\;\ln\left[\int_{Q_{‡}}^{1}e^{-F\left(Q\right)/k_{B}T}dQ/\int_{0}^{Q_{‡}}e^{-F\left(Q\right)/k_{B}T}dQ\right],$$ (2)

where *k*_*B*_ and T are the Boltzmann constant and temperature respectively. *Q*_‡_ is the position of the barrier top in *F*(*Q*), separating the folded and unfolded states and *F*(*Q*) represents the free energy profile on *Q*. Barrier heights Δ*G*_*f*_ were simply defined as Δ*G*_*f*_ = *G*(*Q*_‡_) − *G*(*Q*_u_), where *Q*_u_ is the position of the unfolded state free energy minimum on *Q*.

### Relative contact order

We calculated the relative contact order [49], *RCO*_*K*_ of different circular permutants *K* via

$${\text{RCO}}_{K}=\frac{1}{L\cdot N}\sum_{\left(i,j\right)\in S_{\text{in},K}}\left|i-j\right|,$$ (3)

where *L* is the length of the single domain, and *N* is the total number of the native like contacts (the same for different *K*). *S*_*in*,*K*_ is the contacts set of the circular permutant corresponding to the “central domain” of the misfolded state. Note that the contact order calculation here is using residue-based native contacts (the same ones defined as attractive in the Gō model), instead of all atom native contacts.

### Ising-like theoretical model

An Ising-like model was built based on the native contact map, in which each residue is considered either folded or unfolded and so any individual configuration can be specified as a binary sequence, in a similar spirit to earlier work [50–52]. Interactions between residues separated by more than two residues in the sequence are considered. To simplify the analysis, we also consider that native structure grows only in a single stretch of contiguous native residues (native segment), which means the configurations such as …UFFFUUUUU… or …UUUUUFFFU… are allowed, however, …UFFFUUUFFFU… is not allowed (“single sequence approximation”)[50]. Each residue which becomes native incurs an entropy penalty Δ*S*, while all possible native contacts involving residues within the native segment are considered to be formed, each with a favourable energy of contact formation *ϵ*.

The partition function for such a model can be enumerated as:

$$\begin{array}{ll} Z & =\underset{\chi }{\sum }\text{exp}\left[-\frac{G(\chi )}{k_{\text{B}}T}\right]\\ & =\underset{\chi }{\sum }\text{exp}\left[-\frac{n(\chi )\epsilon -N_{f}(\chi )T\Delta s}{k_{\text{B}}T}\right] \end{array}$$

where *k*_*B*_ and *T* are the Boltzmann constant and temperature. *G*(*χ*) is the free energy determined by the number of native contacts *n*(*χ*) in the configuration *χ*, and the number of native residues, *N*_*f*_(*χ*). The distribution of the microstates (*χ*) can be efficiently generated by the Metropolis-Hastings method with Monte Carlo simulation. In each iteration, the state of one randomly chosen residue (among the residues at the two ends of the native fragment and their two neighbouring residues) is perturbed by a flip, from native to unfolded or from unfolded to native, taking the system from a microstate *χ*_1_ with energy *E*_1_ to a microstate *χ*_2_ with energy *E*_2_. The new microstate is subject to an accept/reject step with acceptance probability

$$P_{\text{acc}}=\min\left[1,\exp\left(-\frac{E_{2}-E_{1}}{k_{B}T}\right)\right].$$ (4)

To mimic the folding stability difference between native and circular permutant folds, a penalty energy term *E*_*p*_ has been added whenever the native fragment crosses the midpoint of the sequence from either side (the function *θ*(*χ*) above is 1 if this is true, otherwise zero). That situation corresponds to formation of a domain-swapped structure, in which there is additional strain energy from linking the termini, represented by *E*_*p*_. We only use the Ising model here to investigate formation of the first domain (either native or circular permutant), by rejecting any proposed Monte Carlo step that would make the native segment longer than the length of single domain, *L*.

## Results

### First passage simulations of misfolding in multidomain proteins

In order to characterize the potential misfolding properties of each type of domain, we have used a Gō-type energy function based on the native structure. Such models have successfully captured many aspects of protein folding, including *ϕ*-values [53, 54], dimerization mechanism [55, 56], domain-swapping [57–60], and the response of proteins to a pulling force [61, 62]. More specifically, a Gō type model was used in conjunction with single-molecule and ensemble FRET data to characterize the misfolded states and misfolding mechanism of engineered tandem repeats of Titin I27 [7, 12]. We have therefore adopted the same model. Although it is based on native-contacts, it can describe the type of misfolding we consider here, which is also based on native-like structure. Note that this model effectively assumes 100% sequence identity between adjacent domains, the scenario that would most likely lead to domain-swap formation. It is nonetheless a relevant limit for this study, as there are examples in our data set of adjacent domains having identical sequences which do misfold (e.g. titin I27) and those which do not (e.g. protein G).

**Fig 3:**
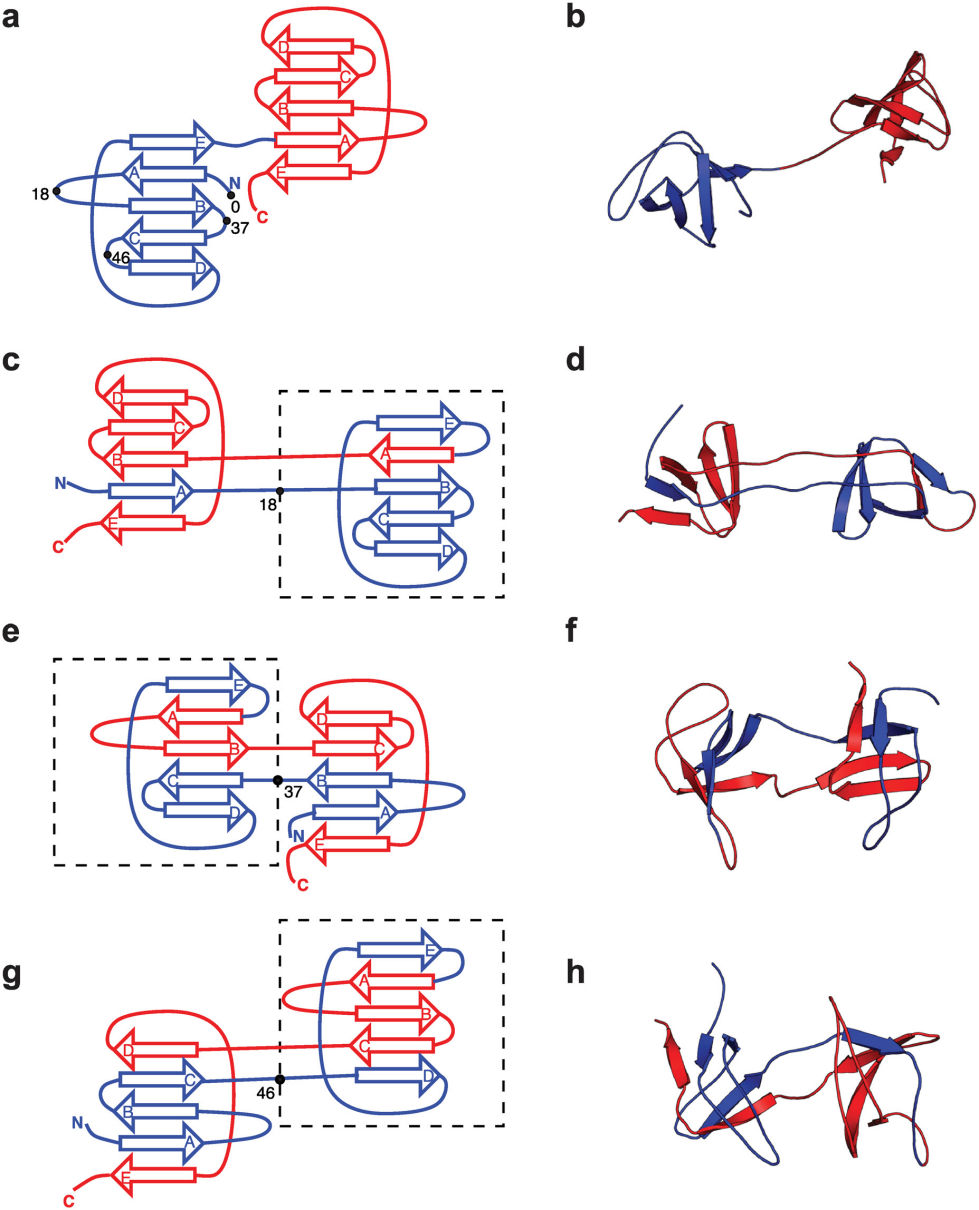
Folded and misfolded topologies of Src SH3. (a) Schematic of Src SH3 fold, in which the three-dimensional *β* sheet structure (shown in (b)) is unrolled into two dimensions, for each domain (N-terminal and C-terminal in blue and red respectively). On the N-terminal domain are indicated the sequence positions *K* ∈ {0, 18, 37, 46} characterizing the possible circularly permuted “central domains”, with *K* = 0 corresponding to the native fold and *K* > 0 indicating the approximate starting residue for the “central domain” misfold. (c), (e), (g): two dimensional representations of the observed misfolded topologies of Src SH3. In each case, the residue *K* characterizing the misfold is indicated by the bullet point and the central domain is enclosed by a broken rectangle. (d), (f), (h): three-dimensional representations of the misfolds shown in (c),(e),(g) respectively.

For each of the folds shown in Fig 2, we ran a large number of simulations, starting from a fully extended, unfolded chain, for sufficiently long (12 *μ*s each) such that the vast majority of them reached either the correctly folded tandem dimer, or a domain-swapped misfolded state similar to that shown in Fig 1e for titin. In fact, for each protein, a number of different misfolded topologies are possible, illustrated for the Src SH3 domain in Fig 3. Each of these domains, shown in conventional three-dimensional cartoon representation in the right column of Fig 3 and in a simplified two-dimensional topology map in the left column, consists of two native-like folded (or misfolded) domains. For convenience, we call the domain formed from the central portion of the sequence the “central domain” and that from the terminal portions the “terminal domain”. We have chosen to characterize each topology in terms of the position, *K*, in sequence after which the central domain begins. Thus, the native fold has *K* = 0, and all the misfolded states have *K* > 0. Typically, because of the nature of domain swapping, *K* must fall within a loop. Of course, there is a range of residues within the loop in question that could be identified as *K* and we have merely chosen a single *K* close to the centre of the loop. This position, and the central domain, are indicated for the Src SH3 misfolded structures in Fig 3. We note that each of these central domains can also be considered as a circular permutant of the native fold, in which the ends of the protein have been joined and the chain has been cut at position *K*.

With this nomenclature in hand, we can more easily describe the outcome of the folding simulations for the seven domain types considered in terms of the fraction of the final frames that belonged to the native fold, versus each of the possible misfolded states. These final populations are shown in Table 1. We see that for five of the domains (SH3, SH2, PDZ, TNfn3, Titin I27), misfolded structures are observed, with total populations ranging from 5–10%. For the remaining two domains, Ubiquitin (UBQ) and protein G (GB1), no misfolded population is observed.

**Table 1:** Summary of misfolding statistics and central domain properties. *K* labels the type of fold/misfold (see text; *K* = 0 is native); RCO is relative contact order [49]. Δ*G*_*f*_ and Δ*G*_*s*_ are the folding barrier and stabilities of a single folded/misfolded domain. Population is frequency of each state at the end of the 1024 trajectories. Maximum standard error on populations is 1.6% for a sample size of 1024. Numbers in brackets are rank correlations with folded/misfolded populations.

Protein	*K*	RCO	Δ*G*_*f*_ (kcal/mol)	Δ*G*_*s*_ (kcal/mol)	Population (%)
SH3	0	0.33	2.7	9.2	95.7
	18	0.38	3.4	2.5	1.1
	37	0.37	4.1	4.8	2.2
	46	0.35	3.0	5.2	1.1
		(-0.63)	(-0.32)	(0.63)	(1.0)
PDZ	0	0.32	2.5	4.5	88.7
	10	0.28	2.5	2.4	6.7
	23	0.33	3.6	1.6	2.3
	43	0.27	2.8	0.3	1.7
	60	0.33	4.2	0.3	0.7
	74	0.26	3.7	0.3	0.0
		(0.32)	(-0.87)	(0.94)	(1.0)
TNfn3	0	0.32	2.4	8.1	89.2
	16	0.33	2.4	1.6	0.0
	28	0.27	3.4	2.8	0.9
	43	0.34	3.9	1.8	2.3
	54	0.29	3.7	1.1	0.6
	66	0.27	3.5	1.8	1.3
	79	0.35	2.5	2.5	5.7
		(0.34)	(-0.11)	(0.74)	(1.0)
UBQ	0	0.29	2.5	4.2	100.0
	9	0.29	3.1	-2.9	0.0
	21	0.28	2.7	-3.2	0.0
	36	0.28	6.3	-6.3	0.0
	61	0.26	3.5	-3.3	0.0
SH2	0	0.24	2.6	6.1	91.7
	11	0.25	3.1	3.1	0.4
	24	0.30	3.2	1.8	0.0
	37	0.28	2.7	3.3	0.9
	49	0.25	3.3	3.2	1.1
	61	0.26	3.8	3.5	2.8
	72	0.27	3.9	2.5	2.6
	89	0.26	3.2	2.0	0.4
		(-0.50)	(0.10)	(0.81)	(1.0)
Titin I27	0	0.34	2.5	8.1	92.0
	16	0.36	3.0	1.5	0.3
	28	0.30	2.8	3.0	3.1
	37	0.33	2.8	2.9	2.9
	53	0.36	3.0	2.0	0.2
	64	0.30	3.0	1.0	0.4
	76	0.33	2.8	2.3	2.0
		(-0.45)	(-0.92)	(0.86)	(1.0)
GB1	0	0.35	2.5	3.1	100.0
	12	0.36	4.4	-5.2	0.0
	23	0.31	4.6	-5.3	0.0
	41	0.27	4.9	-5.4	0.0
	50	0.36	5.3	-5.7	0.0

### Consistency with existing experimental data

The ability to capture domain-swapped misfolds with simple coarse-grained simulations potentially allows us to investigate the origin of the misfolding, and its relation, if any, to the topology of the domain in question. However, we also need to benchmark the accuracy of the results against experiment as far as possible, in order to show that they are relevant. There are two main sources of information to validate our results. The first is the overall degree of domain-swapped misfolding for those proteins where it has been characterized, for example by single molecule AFM or FRET experiments. Qualitatively we do observe good agreement, where data is available: in experiment, domains which have been shown to misfold are TNfn3 (AFM) and Titin I27 (AFM, FRET), which are both found to misfold here, while there is no detectable misfolded population for protein G (AFM), again consistent with our results. We also do not observe any misfolding for Ubiquitin, consistent with the lack of experimental evidence for fully domain-swapped species for this protein [15–23].

Quantitatively, the fractional misfolded population is also consistent with the available experimental data. For instance, the frequency of misfolded domains in native tenascin is ∼ 4% as shown by previous AFM experiments [8], the misfolded population of I27 dimers is ∼5% in single-molecule FRET experiments [7] while the misfolded population of GB1 domains in polyproteins (GB1_8_) is extrememly low (< 0.2%) [14]. Even though the observed misfolding population of the misfolded tandem dimer is low, it is potentially a problem considering that many of the multidomain proteins in nature have large number of tandem repeats, such as Titin which contains twenty-two I27 repeats [63]. Recent FRET experiments on I27 tandem repeats have shown that the fraction of misfolded proteins increases with the number of repeats. For the 3- and 8-domain polyproteins, the fraction of misfolded domains increases by a factor of 1.3 and 1.8, respectively, relative to a tandem dimer [12].

**Fig 4:**
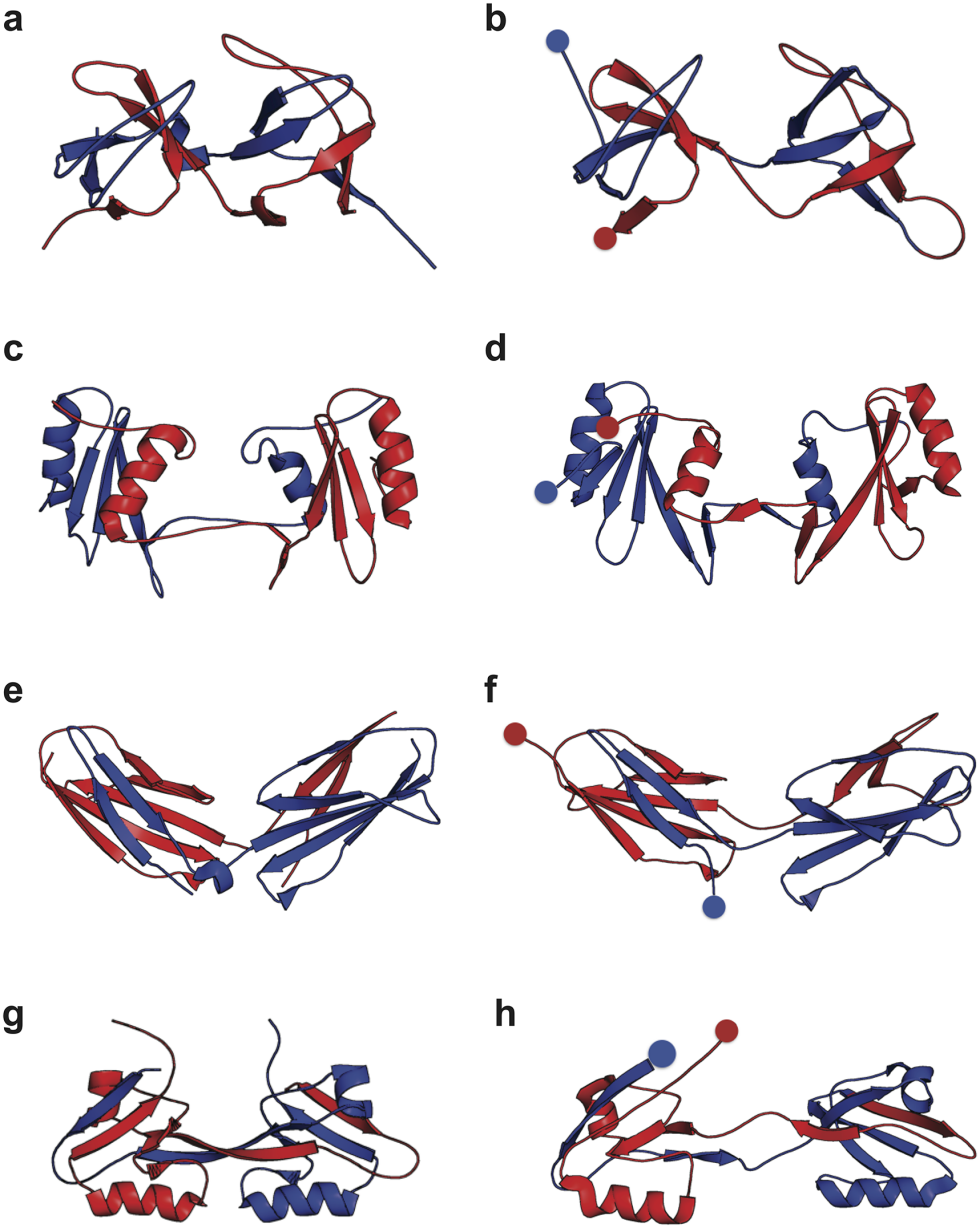
Comparison of domain-swapped misfolds with experimental structures. Selected misfolded dimeric tandems obtained from the simulations (right column) are compared with corresponding experimental structures (solved by crystallography or NMR) of domain-swapped dimers involving two separate protein chains (left column). The proteins are, from top to bottom (a),(b): SH3, (c),(d): SH2, (e),(f): TNfn3 and (g),(h): PDZ domains The PDB accession codes are 1I07, 1FYR, 2RBL and 2OSG respectively.

The second type of evidence comes from experimental structures of domain-swapped dimers. For several of the proteins, bimolecular domain-swapped structures have been determined experimentally. While no such structures have yet been determined for single-chain tandem dimers, we can compare the misfolded states with the available experimental data. For each experimental example, we are able to find a corresponding misfolded species in our simulation with very similar structure (related by joining the terminis of the two chains in the experimental structures). The domain swapped dimers solved obtained from experiments (Fig 4a, 4c, 4e and 4g) are strikingly similar to the domain swapping dimeric tandem from simulations, which are the domain swapped SH3 domains when *K*(sequence position after which the central domain begins) = 37 (Fig 4b), SH2 with *K* = 72 (Fig 4d), TNfn3 with *K* = 28 (Fig 4f) and PDZ with *K* = 23 (Fig 4h). Most of these states have relatively high population among all the possible misfolds as observed from the simulations (“Population” in Table 1). While the coverage of possible domain swaps is by no means exhaustive, the observed correspondence gives us confidence that the misfolded states in the simulations are physically plausible.

### Circular permutants as models of misfolding intermediates

Having shown that the misfolding propensities we obtain are qualitatively consistent with experimental evidence (and in the case of Titin I27, in semi-quantitative agreement with single-molecule FRET), we set out to establish some general principles relating the properties of each domain to its propensity to misfold in this way. We can start to formulate a hypothesis based on the alternative folding and misfolding pathways illustrated in Fig 1. Native folding has as an intermediate a state in which either the N- or the C-terminal domain is folded. In contrast, on the misfolding pathway, the first step is formation of the central domain, followed by that of the terminal domain. This parallel pathway scheme suggests that a descriptor of the overall misfolding propensity may be obtained from the rate of formation of a single correctly folded domain, relative to that of the central domain (neglecting back reactions, because this are rarely seen in our simulations). We can study the central domain formation in isolation, since these structures are just circular permutants of the native fold, i.e. the two proteins have the same sequence as the native, but with the position of the protein termini moved to a different point in the sequence, as is also found in nature [35]. These structures can be thought of as originating from the native by cutting a specific loop connecting secondary structure elements (the free energy cost of splitting such an element being too high), and splicing together the N- and C- termini. In the context of the tandem dimers, the position at which the loop is cut is the same *K* that defines the start of the central domain in sequence.

We investigate the role of the central domain by characterizing the free energy landscape of the single domain of each system, as well as all of its possible circular permutants, using umbrella sampling along the reaction coordinate *Q*_*K*_. *Q*_*K*_ is exactly analogous to the conventional fraction of native contacts coordinate *Q* [64], but defined using the corresponding (frame-shifted) contacts in the circular permutant pseudo-native structure. The index *K* indicates the position along the sequence of the WT where the cut is made in order to convert to the circular permutant.

**Fig 5:**
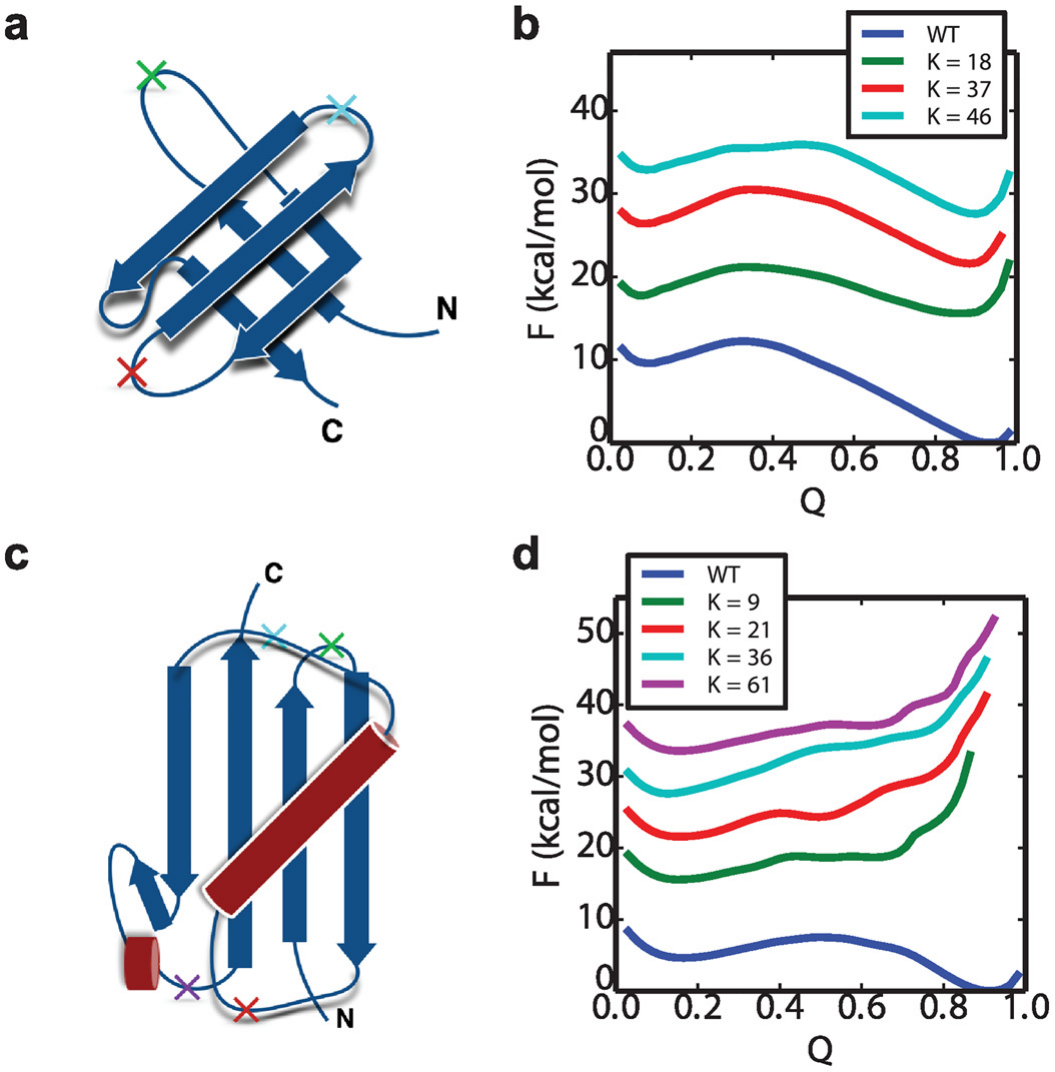
Free energy profile of WT and its circular permutant domains. The structures of SH3 and Ubiquitin are shown in (a) and (c), with the “cut” positions *K* in the WT to form circular permutant labeled with crosses. (b) is the free energy surfaces *F*(*Q*) of WT SH3 as well as its circular permutants at 300K. (d) is the *F*(*Q*) of WT Ubiquitin and its circular permutants at 290K. The labels *K* indicate the residue index of the cut position. The free energy curves of the circular permutant cases are shifted vertically for visual clarity, and coloured using the colours corresponding to the crosses in (a) and (c). The free energy plots of the other systems: GB1, SH2, TNfn3 and PDZ are shown in Fig A in S1 Text.

The free energy surfaces *F*(*Q*_*K*_) of two representative systems, SH3 and Ubiquitin, are shown in Fig 5, with the data for the remaining proteins given in the Fig A in S1 Text. The free energy barrier height for folding Δ*G*_*f*_ and the stability Δ*G*_*s*_ are listed in the Table 1. The free energy plots indicate that the single domains of Ubiquitin and GB1 are stable only for the native sequence order, and not for any of the circular permutants. Based on the type of misfolding mechanism sketched in Fig 1, one would expect that unstable circular permutants would result in an unstable central domain, and consequently no stable domain-swappping misfolding would occur in the dimer folding simulations, as we indeed observe. This is also consistent with previous studies of polyproteins of GB1 and Ubiquitin using using AFM experiments, which reveal high-fidelity folding and refolding [14, 65, 66]. We note that only under very strongly stabilizing conditions is any misfolding observed for ubiquitin dimers: running simulations at a lower temperature (260 K), we observe a very small (1.3%) population of misfolded states from 1024 trial folding simulations. At a higher temperature of 295 K, once again no misfolding is observed.

In contrast to the situation for GB1 and Ubiquitin, all of the circular permutants of the SH3 domain in Fig 5 are in fact stable, although less so than the native fold. The destabilization of circular permutants relative to native is in accord with the experimental results for the Src SH3 domain [26] (rank correlation coefficient stabilities is 0.80). The other domains considered also have stable circular permutant structures. This is consistent with the fact that all of these domains do in fact form some fraction of domain-swapped misfolded states. The simplest view of the misfolding mechanism would be as a kinetic competition between the correctly folded intermediates versus the domain-swapped intermediates with a central domain folded (i.e. a “kinetic partitioning” mechanism [67]). In this case one might naively expect that the propensity to misfold would be correlated with the relative folding rates of an isolated native domain and an isolated circular permutant structure. However, the folding barriers Δ*G*_*f*_ projected onto *Q* (for native) or *Q*_*K*_ (for circular permutants) show little correlation to the relative frequency of the corresponding folded or misfolded state, when considering all proteins (Table 1). Since this barrier height may not reflect variations in the folding rate if some of the coordinates are poor (yielding a low barrier) or if there are large differences in kinetic prefactors, we have also directly computed the folding rate for the circular permutants of those proteins which misfold, and confirm that the rates of formation of the native fold and circular permutants are similar. We indeed obtain a strong correlation between the folding rate of the isolated circular permutant and the folding barrier Δ*G*_*f*_ (Table B in S1 Text), which implies Q is a sufficiently good reaction coordinate here. We have also considered the relative contact order (RCO) as a proxy for the folding rate, since it has been found to correlate with folding rates for two-state folding proteins [49, 68]. However, the RCO calculated based on the native or circularly permuted folds did not correlate with either the barrier height for single domain or circular permutant folding, or with the extent of misfolding in dimeric tandem proteins (Table 1). Since the folding rates do not explain misfolding propensities by themselves, another possibility is that the reverse reactions have to be considered. However, once they had formed, in most cases we did not observe unfolding of the first native domain, or of the intermediate with central domain folded, indicating that back reactions should not be needed, at least to explain the simulation data. This lack of refolding is a consequence of the significant stability of the native folds, which controls the relative folding and unfolding rates (and indirectly, those of the circular permutants). Under these conditions, given that folding rates are much higher, once a native fold (or circular permutant misfold) has formed, it is much more likely that a second domain will fold, rather than the first domain unfolding. Our choice of stabilizing conditions was motivated by the fact that misfolding is observed in experiment under conditions where the folded state is much more stable, and the stabilities (Δ*G*_*s*_) of the folded single domains in our simulations are generally comparable to those in experiment (experiment vs simulation, UBQ: 6.1 vs 4.2, GB1: 5.3 vs 3.1, PDZ: 7.5 vs 4.5, SH3: 4.1 vs 9.2, Titin: 7.5 vs 8.1, Tnfn3: 5.3 vs 8.1 kcal/mol) [26, 69–72].

**Fig 6:**
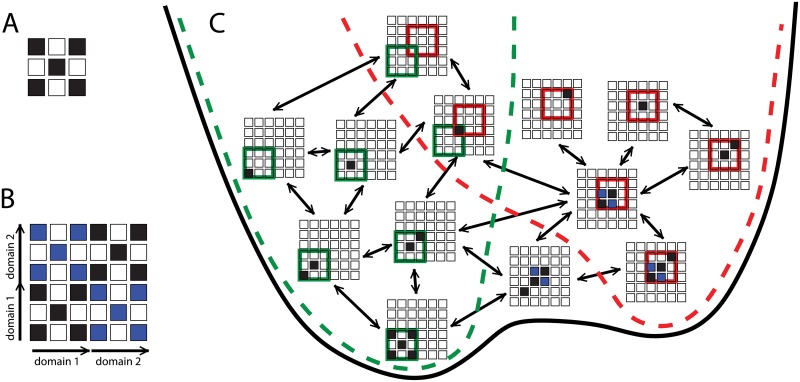
Folding/misfolding funnel. Illustration of relation between folding funnels for native and domain-swapped domains. (A) Example native contact map, highly coarse-grained for simplicity. (B) Map of all possible native-like contacts for a two-domain protein, showing native contacts in black and domain-swapped contacts in blue. (C) In the context of the two-domain sequence, the folding funnels for a single native domain (green broken line) and domain-swapped domain (red broken line) are interconnected, forming part of a single global funnel (black line). States are considered part of the native funnel if all contacts formed belong to the native state, and to the domain-swap funnel if all contacts formed belong to the domain-swapped structure. Note that only a subset of possible states are shown, for clarity (e.g. other domain-swapped species are possible). Only states with a single native-like stretch of residues are considered, whose length does not exceed that of a single folded domain. Arrows connect states differing by a single coarse grained residue flipping between native and non-native.

On the other hand, we did note that there was a significant, and unexpected, correlation between the population of the final folded or misfolded states and the *stability* Δ*G*_*s*_ of the corresponding intermediate. Spearman rank correlation coefficients between the folded stability Δ*G*_*s*_ of the intermediate structure and the frequency of folded/misfolded states were 0.63, 0.94, 0.74, 0.81, 0.86 for the SH3, PDZ, TNfn3, SH2 and Titin I27 domains respectively. We note that there is also a reasonable correspondence between the relative stabilities of circular permutants in simulation and experiment, where data are available [12, 26]. How can the correlation with stabilities rather than folding rates of the isolated domains be understood? The resolution lies in the difference between the folding to either type of intermediate represented in Fig 1, and folding of the single domain “models” for these species, namely that the intermediates fold *in the context of the full sequence*. This is important because a large fraction of native (or native-like) contacts are shared between the native fold and the various misfolded domains. As such, the native and misfolded states can be considered as belonging to the same folding funnel, with differentiation between the two occurring at a late stage of folding. This scenario is illustrated schematically in Fig 6, in which folding to either a state with one native domain folded (on left), or one possible domain-swapped misfolded intermediate (on right) are considered. The states of the proteins are represented by very coarse-grained contact maps (e.g. representing contacts between pairs of *β*-strands [73], rather than between residues). As can be seen, dividing this funnel into the separate funnels by considering only native contacts for the native or circularly permuted fold would be misleading (green and red funnels respectively), since the two funnels share several configurations, and many of their states can be converted to one in the other funnel by flipping a single coarse-grained “residue” between folded and unfolded states.

**Fig 7:**
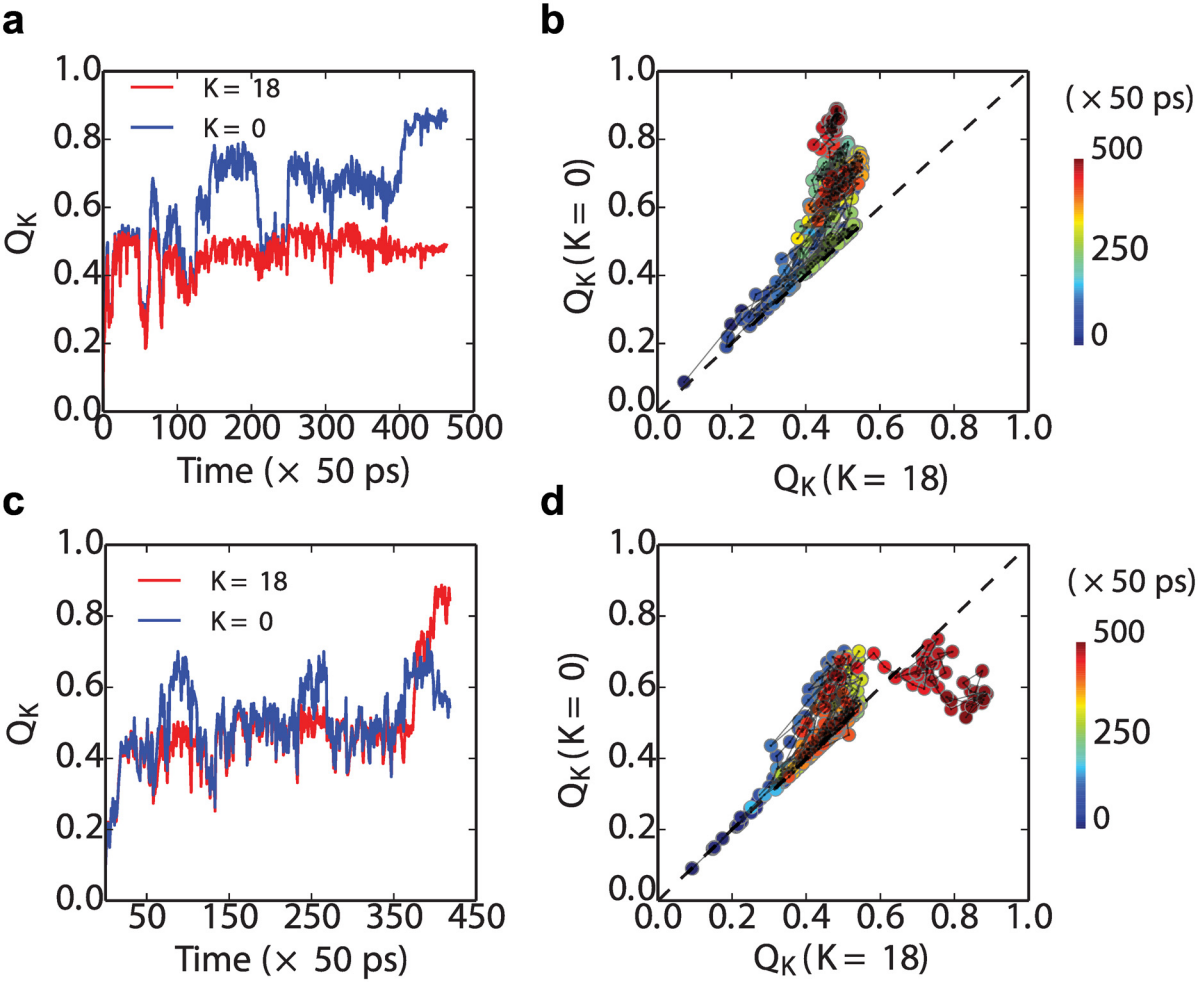
Transition paths for the formation of the first (folded or misfolded) domain in tandem SH3 dimers. Folding (a) and misfolding (c) kinetics are projected along the reaction coordinate *Q*_in_, where two different kinds of *Q*_in_ are chosen depends on *K*, which is for the native fold and the circular permutated misfold when *K* = 0 and 18, respectively. Transition-path segments are defined as being between *Q*_*K*_ = 0.1 and *Q*_*K*_ = 0.9. In the right panels, the same trajectories are projected onto the *Q*_*K* = 0_ and *Q*_*K* = 18_ (panel (b) for trajectories in (a) and (d) for those in (c).

We can see this explicitly by plotting some representative folding transition paths from the Src SH3 dimer simulations. In Fig 7 top row, we show a folding event for a simulation which forms a native fold (at the N-terminus), and in the bottom row, for a simulation which forms a circularly permuted central domain with *K* = 18. Each event is projected onto two different reaction coordinates,*Q*_*K*_, for *K* = 0 (standard native *Q*) and *K* = 18 (the *Q* when the circular permutant for *K* = 18 is considered as “native”). As is evident, a large fraction of the transition path looks very similar in Fig 7b and 7d, with contacts that could be considered equally as native-like or central domain-like being formed initially in the lower left part of each plot. Around *Q*_0_ ≈ *Q*_18_ ≈ 0.5, the first trajectory moves toward the native structure, where it terminates (Fig 7b). The second trajectory also deviates initially more toward the folded structure, but then switches back near the end to form the central domain structure instead (Fig 7d). A similar branching of folding pathways has also been proposed in a recent computational study of domain swapped dimer formation [74].

The common funnel picture helps to explain why the stability of the isolated native or circular permutant domains may be correlated with their frequency of formation in the context of the full length sequence in which either could potentially be formed. Initially, nucleation of folding could occur by formation of native contacts anywhere in the sequence. Indeed, they are most likely to form near the centre of the chain. However, as more native/native-like structure is accumulated, the nascent, partially folded protein will be biased to form the contacts leading to the lower free energy structure, and so the folding nucleus is likely to move towards one of the termini of the protein. We note that while a previous study suggested that the stability of the individual domains might be affected by conjugation to another folded domain [75], this is unlikely to be relevant because in our case the misfolding is controlled by formation of the first domain, while the second domain is still unfolded.

**Fig 8:**
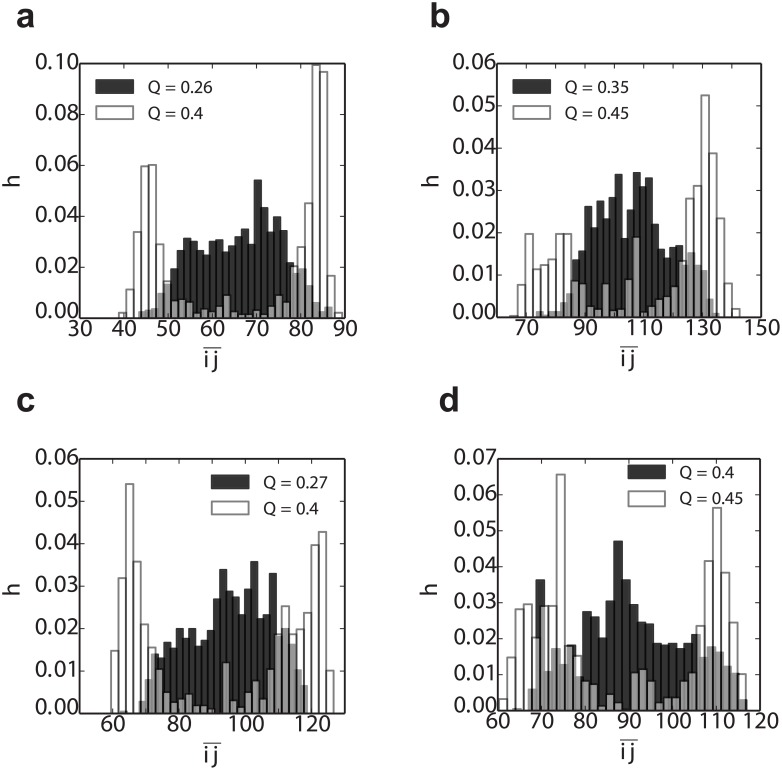
Distribution of the “folding nucleus” location $p(\bar{ij}|Q)$ from the tandem dimer simulations (Table 1). The $p(\bar{ij}|Q)$ of the (a) (SH3)_2_ (b) (SH2)_2_ (c) (TNfn3)_2_ and (d) (PDZ)_2_ are extracted at two different Q on the folding pathway (see individual figure legends for *Q* values). Note that the Q ∼ 0.5 corresponds to the structure with the first domain fully formed. The spread of contacts in sequence, within a given conformation, also becomes narrower with increasing Q (Fig B of S1 Text).

Further insight into how the above free energy bias influences the outcome of the folding kinetics can be obtained by considering the progressive formation of folded structure. In order to characterize the location of nascent folded structures, we define a new order parameter $\bar{ij}$ representing the average position of native contacts along the sequence,

$$\bar{ij}\left(\chi \right)=\frac{1}{\left|S(\chi )\right|}\sum_{\left(i,j)\in S(\chi \right)}\frac{i+j}{2},$$ (5)

where (*i*, *j*) is the native or native-like contact formed by the residues *i* and *j* in the configuration *χ*, and *S*(*χ*) is the set of all such contacts which are formed in *χ*. We can locate the position of nascent structure in the sequence by plotting the distributions of $\bar{ij}\left(\chi \right)$ for *χ* drawn from the equilibrium distribution at selected values of the global coordinate *Q*, defined as the fraction of native contacts in the native dimer structure (i.e. *Q* = 0.5 corresponds to a single folded or misfolded domain; both native and native-like contacts are counted, and divided by the total number of contacts in the native state). Fig 8 shows that early in folding, at low *Q* values (shaded histograms in Fig 8), the distribution of $\bar{ij}$ is broad, and centered in the middle of the sequence. This implies that folding could potentially begin at many positions along the sequence, with no initial preference for folded or circularly permuted structure. However, as folding proceeds closer to formation of a complete domain, $\bar{ij}$ develops two maxima, one in the N-terminal and one in the C-terminal part of the chain, corresponding to native domain formation. The nascent native-like structure thus naturally migrates towards the termini to avoid the free energy penalty of forming a circularly permuted misfolded intermediate.

### Ising-like theoretical model

The results from the previous sections show that the misfolding propensity is highly correlated with the the stability of the isolated native domain and its circular permutants. To further explain how this might occur, we investigate the dependence of the misfolding propensity on the stability of the central domain in the context of full sequence (dimeric tandem). We have constructed an even simpler simulation model for formation of the first intermediate (native, or circularly permuted), by using a simulation of a Wako-Saito-Muñoz-Eaton Ising-like model [51, 76]. In the version we consider here, each residue is considered either to be folded or unfolded, so that each configuration can be described as a binary string. Furthermore, we impose the single-sequence approximation, namely that all native-like structure forms in a single segment of contiguous residues. We also restrict the number of folded residues to be at most one half of the dimer sequence length, so that only a single folded or misfolded domain can form, the aspect we are most interested in. To model the stability difference between native and circularly permuted domains, we introduce an additional energy penalty *E*_*p*_ for any folded segment which crosses the midpoint of the dimer sequence. Such a folded segment must be forming a circular permutant misfold and as such will incur some additional “strain” energy from joining the termini of the original fold.

**Fig 9:**
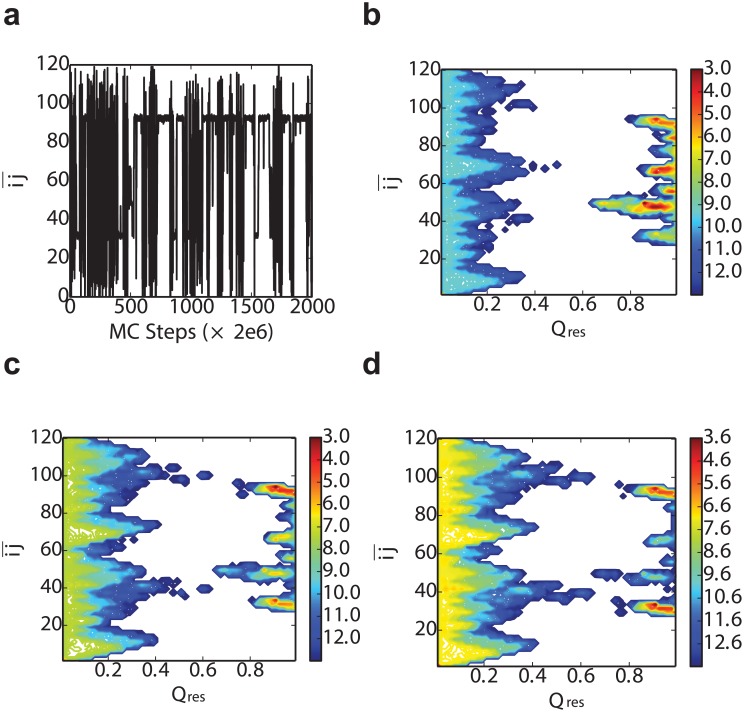
Ising-like model. a) Monte Carlo simulation trajectory segment when *E*_*p*_ = 4.0 kcal/mol. The free energy profile of $\bar{ij}$ vs *Q*_res_ changes when the difference of the stability between the WT and the circular permutant become larger and larger, in which cases the *E*_*p*_ are b) 0.0, c) 4.0 and d) 6.0 respectively. All the free energy plots are at the temperature *T* = 525 K.

We show results from a typical Monte Carlo trajectory for this model in Fig 9. We have used two parameters to characterize the results, the fraction of native or native-like contacts, *Q*_res_, and $\bar{ij}\left(x\right)$ (Eq 5). *Q*_res_ equals to the number of residues which are in the native-like state divided by the total number of residues of one domain (*L*). The projection of a trajectory for the model onto $\bar{ij}$ in Fig 9a shows that the most stable states occur for $\bar{ij}$ in the center of either the first of the second natively folded domain. Nonetheless, there are other stable states at intermediate $\bar{ij}$, which correspond to the circular permutant intermediates. These have a lower stability, because a value *E*_*p*_ > 0 was used in this instance. The effect of the stability penalty for the circular permutants is illustrated by the two-dimensional free energy surfaces $F(Q_{\text{res}},\bar{ij})$ in Fig 9b–9d. In all cases, there are minima at low *Q*_res_ corresponding to unfolded structures and at high *Q*_res_ for folded (native or circular permutants). If the penalty *E*_*p*_ = 0 (Fig 9b), in addition to the stable native folds at $\bar{ij}∼30$ and $\bar{ij}∼90$, there are a variety of other free energy minima at high *Q*_res_ corresponding to circular permutants, which have essentially identical free energy to the native fold. However, as *E*_*p*_ is increased, the relative population of these misfolded states decreases (Fig 9c and 9d), as expected.

### Predicting circular permutant stability using alchemical free energy method

**Fig 10:**
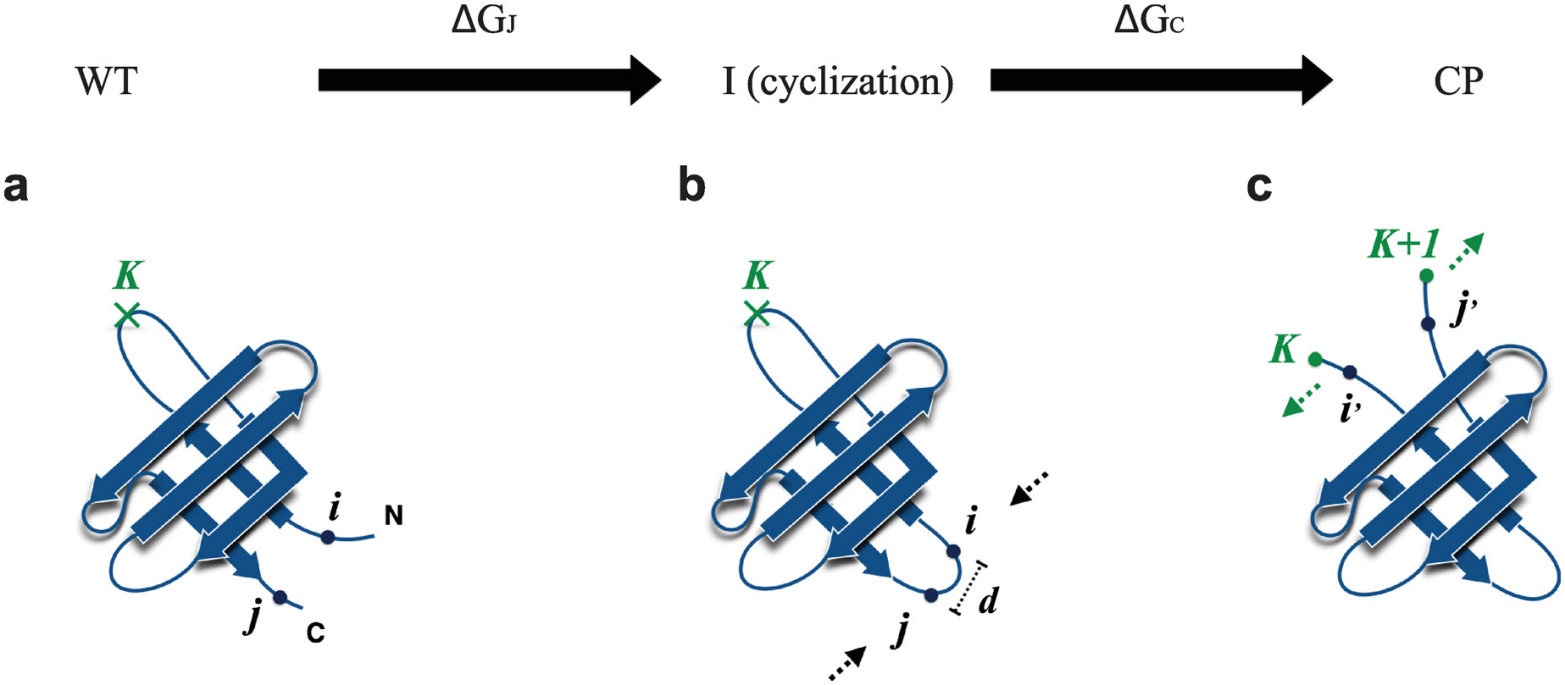
Alchemical transformation from native to circular permutant. (a) Native structure; (b) cyclized structure; (c) circular permutant after cutting another loop.

Knowing that misfolding correlates with the relative stability of the native single domain and its circular permutants is useful because it suggests a means to predict the likelihood of misfolding, provided one has an estimate of the circular permutant stability. While one could attempt to determine this experimentally, by synthesizing the circular permutants, or computationally, as we have done, it would be very helpful to have a quick method to estimate this stability *a priori* [77]. Here, we have developed such a method, based on an alchemical transformation from the native to the domain-swapped misfolded state: the overall conversion between the native and circular permutant can be expressed as the sum of the free energy changes of two steps as shown in Fig 10: firstly, joining the N- and C- termini of WT (Fig 10a) to form a cyclic intermediate state (I) (Fig 10b), in which procedure the free energy change is (Δ*G*_J_). Note that this step is the same for all circular permutant folds. The second step is to cut different loops on the cyclic configuration I to form a circular permutant CP (Fig 10c) with the change of free energy (Δ*G*_C_):

Assuming the native and circular permutant unfolded states to have the same free energy, the overall change in stability between the circular permutant (CP) and the wild-type (WT) is:

$$\begin{array}{ll} \Delta \Delta G_{\text{tot}} & =\Delta G_{\text{CP}}-\Delta G_{\text{WT}}\\ & =\Delta G_{\text{J}}+\Delta G_{\text{C}}. \end{array}$$

For the first step (Fig 10a and 10b), in order to join the two termini, they must be sufficiently close. In general, bringing them closer together will require peeling off a small part of the native structure, starting from the termini. If we imagine that all of the structure between residues *i* at the N-terminus and *j* at the C-terminus remains native, then the change in free energy for linking the termini for cyclization, Δ*G*_J_, can be split into energetic and entropic components:

$$\Delta G_{\text{J}}=\left(E_{\text{I}}-E_{\text{WT}}\right)-T\left(S_{\text{I}}-S_{\text{WT}}\right).$$

Assuming the states of the residues *p* ∈ {0 .. *i* − 1} and *q* ∈ {*j* + 1 .. L}, which are on the N- and C- termini respectively, change from the native state to non-native state (joint loop), the total energy increase will be ∑_*x* ∈ *σ*(*p*)_ *ϵ*_*px*_ + ∑_*x* ∈ *σ*(*p*)_ *ϵ*_*qx*_, which is the summation of all the native contact energy, in the Go model over the sets of residues *σ*(*p*), *σ*(*q*) involving residues *p* and *q* respectively. *x* represents the residues that form the native contacts with either *p* or *q*. We approximate the entropy gained per residue as *δs*, where *δs* = ∑_native(*i*,*j*)_ *ϵ*_*ij*_/(*TN*), where *N* is the number of residues and *T* is the folding temperature. The gained entropy is set to 0 if residue *p* or *q* does not have any contact with other part of the protein except for the neighboring residues, and the number of such residues is denoted by *κ*. The average length contribution (*r*_0_) of peeling off each residue from the native structure is set to 3.5 Å here. The topological requirement of joining the two termini by peeling off residues 1 to *i* − 1, and *j* + 1 to L from the native state is that the linear distance between the residues *i* and *j* (*d*(*R*_*i*_,*R*_*j*_)) on the native structure is shorter than the effective length contributed by the joint parts:

$$d\left(R_{i},R_{j}\right)<\left(i+L-j-M\right)r_{0}$$ (6)

Note that if N- and C- termini point in opposite directions, such as the TNfn3, Titin I27, UBQ and GB1 domains (Fig 2), around six residues (three on each side) of the two termini will form the turn of the joint loop which does not contribute to the the effective length. Therefore, an offset number M = 6 is used in this case. This is justified because turns in proteins are usually defined by four residues (or 3 residue-residue bonds) [78]. For SH3, SH2 and PDZ domains (Fig 2), whose N- and C- termini align to the same direction, M is set to 0. With the above condition (Eq 6), the minimum overall change of Δ*G*_J_ by adjusting *i* and *j* could be given by:

$$\Delta G_{\text{J}}=\min_{i,j}\left\{\sum_{p\in 0,..,i-1}\epsilon \left(p,x\right)+\sum_{q\in j+1,..,N}\epsilon \left(q,x\right)-T\left(i-1+L-j-\kappa \right)\delta s\right\},$$

where

i∈{0,1,..,9}andj∈{L-9,..,L}

Analogously, for the second step (Fig 10b and 10c), assume the loop is cut at the position between residue position *K* and *K* + 1, the states of the residues on each side of the cutting point *p*′ ∈ {*i*′,.., *K*} and *q*′ ∈ {*K* + 1,.. *j*′}, will change from the native state to the non-native state. The gained entropy per residue is *δs*. ∑_*x* ∈ *σ*(*p*′)_ *ϵ*_*p*′*x*_ and ∑_*x* ∈ *σ*(*q*′)_ *ϵ*_*q*′*x*_ are the summation of all the energy of the native contacts which are broken due to the cutting. Therefore, by comparing different combinations of *i*′ and *j*′, the minimum change of stabilities Δ*G*_C_ in this step is:

$$\begin{array}{ll} \Delta G_{C} & =(E_{C}-E_{I})-T(S_{C}-S_{I})\\ & =\underset{i^{\prime },j^{\prime }}{\text{min}}\{\underset{p^{\prime }\in i^{\prime }+1,..,K}{\sum }\epsilon (p^{\prime },x)+\underset{q^{\prime }\in K+1,..,j^{\prime }-1}{\sum }\epsilon (q^{\prime },x)-T(j^{\prime }-i^{\prime }-1)\delta s\}, \end{array}$$

where

$$i^{\prime }\in \left\{K-3,..,K\right\}\;\text{and}\;j^{\prime }\in \left\{K+1,..,K+4\right\}.$$

**Fig 11:**
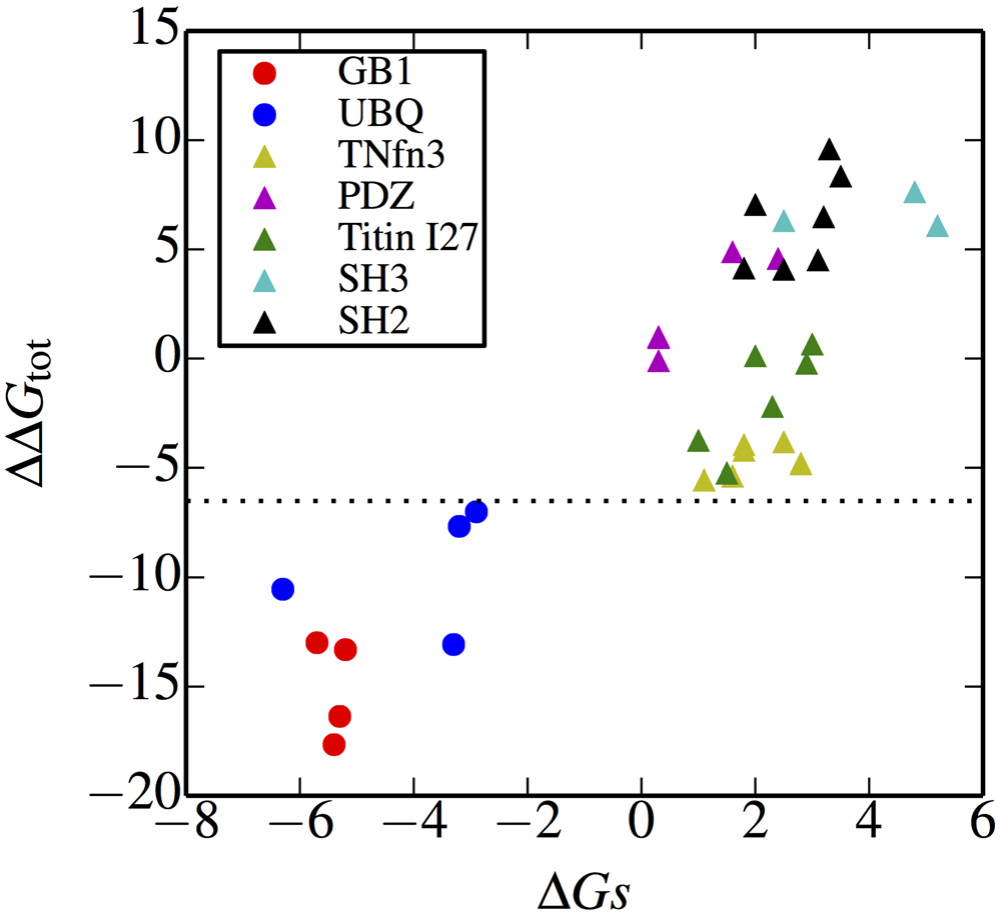
ΔΔ*G*_tot_ from alchemical model vs Δ*G*_*s*_ (Table 1).

The ΔΔ*G*_tot_ calculated using this alchemical free energy method is very well correlated with the stability of the circular permutant Δ*G*_*s*_ obtained by umbrella sampling simulations (Table 1) as shown in Fig 11. It is consistent with the experimental results that GB1 and Ubiquitin have the most unstable circular permutant folds in general. The main contribution of ΔΔ*G*_tot_ is from Δ*G*_J_, since the enthalpy penalty is large when many native contacts are broken by joining the terminis such as in the case of UBQ and GB1. The free energy cost of cutting the loop (Δ*G*_c_) is relatively small and is similar for all the circular permutants during the transformation (Fig 10).

### Effect of linker length

**Fig 12:**
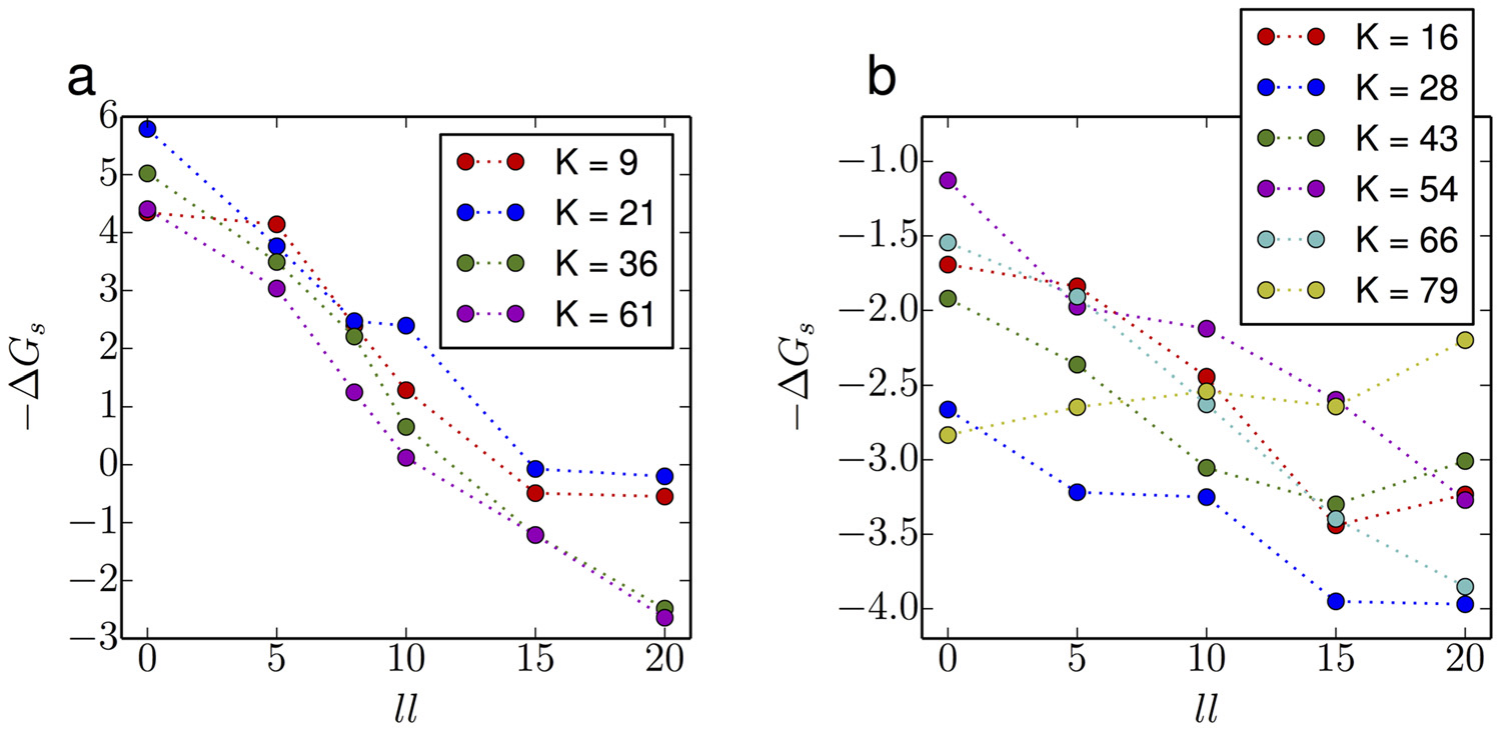
Stability of different CP of Ubiquitin (a) and TNfn3 (b) with different linker lengths. The linker sequence composition is (GS)_2_-S, (GS)_5_, (GS)_7_-S and (GS)_10_ giving *ll* of 5, 10, 15 and 20 respectively.

From the alchemical method we can see that the difference of stability ΔΔ*G* largely depends on the native contacts that are broken in the procedure when joining the N- and C- termini. However, ΔΔ*G* could also be lowered by extending the linkers between domains, for instance, by adding extra residues at the two termini. If the loop formed by the linker is long enough, fewer native contacts will need to be disrupted, so that the circular permutant folds would be more stable. Therefore we have investigated the stability of circular permutant folds as a function of the length of C-terminal extension, by adding Gly-Ser repeats (forming no native contacts). This extra peptide corresponds to the linker between the tandem domains. The stability of circular permutants, obtained from simulations using umbrella sampling, as a function of linker length (*ll*) is shown in Fig 12 (raw potentials of mean force on *Q*_*K*_ in Figs C and D in S1 Text). As expected, longer linkers between the tandem repeats give more stable circular permutants. The relative change from *ll* = 0 to *ll* = 20 is roughly the same for all circular permutants of a given protein, as expected since the change in all cases is the same loop extension. Note, however, that the effect is much larger for ubiquitin than for TNfn3. To investigate the consequences of the change of central domain stability for the misfolding propensity of tandem repeats, we carried out first passage simulations of a tandem dimer of Ubiquitin with linker lengths of 5,8 and 10 residues respectively. The setup of the dimer simulations was the same previously. For each linker length, 1024 independent simulations were run from fully extended structures. No domain-swapped misfolding was found for *ll* = 5 and *ll* = 10, however, we indeed obtained three domain-swapped misfolding events for *ll* = 8. Two of the misfolds belong to the *K* = 61 (Fig 12a) type and the other one is *K* = 36 type domain-swapping. As one can see from Fig 12a, the circular permutants *K* = 36 and *K* = 61 are the ones which are most stable with *ll* = 8. However, they are still somewhat unstable, explaining the small fraction of misfolded states obtained. In this case it is clear that the length of the linker between the termini of ubiquitin is one way in which domain-swapped misfolding is avoided in this protein: since ubiquitin is synthesized initially as an N-C linked polyubiquitin chain [40], it is essential to avoid such misfolding, given the importance of this protein to cellular homeostasis. It should be noted though that the influence of the linker depends very much on the protein, as might be expected, from the much smaller effect on the stability of circular permutants of TNfn3 than those of ubiquitin in Fig 12. In experiments on titin I27, the misfolded population was, within error, the same with and without the addition of a four residue RSEL linker [7].

Lastly, we comment briefly on the effect of linker composition. Although we treat the linkers as structureless chains, not forming native contacts, there may be some effect of linker flexibility, arising from the backbone dihedral potential in our model. To test for this effect, we have carried out an additional 1024 independent simulations with the dimeric tandem repeat of the SH3 domain using a different four residue linker composition, GGGG, rather than the original DETG, as used in the original circular permutant studies by Serrano *et al* [26]. With the new linker GGGG, the observed misfolded populations are 94.3%, 1.3%, 2.3% and 2.1% for K = 0, 18, 37 and 46 respectively. The differences are not statistically significant compared to the results with the original linker.

### Conclusions

We have investigated the factors which favour formation of domain-swapped misfolded states in multidomain proteins, by building on knowledge of the folding/misfolding mechanism. Counter to our original expectations, the misfolding yield does not depend primarily on the relative folding rates of the native single-domain protein and its circular permutants, representing intermediates for correct folding and misfolding respectively. Although the folding rates of wild-type and circular permutants may often be quite similar, the fraction of misfolded protein is much smaller than this comparison would suggest. Instead, it appears that misfolding is correlated with the stability of the native single-domain protein relative to its circular permutants. This can be understood because the rate of formation of the first intermediate (native-like or misfolded) occurs in the background of the full-length sequence. In this context, while folding may be initiated at any point in the chain, the nascent structure will tend to migrate towards the N- or C-terminus because of the free energy bias towards the native fold; circular permutants invariably pay a cost in stability for joining the protein termini. Thus the folding rate of isolated circular permutants relative to wild-type protein may not be a good proxy for these rates in the context of the full length sequence, whilst the domain stability is a better guide as to the free energy bias towards a particular structure. This suggests that the rates of formation of these domains inferred from single-molecule experiments [12] should be interpreted as the rates in the context of the full length sequence. In our analysis, we have neglected the effect of back-reactions. Since these occurred rarely in the simulations, they were not needed to explain the results. We have also quantified the effect of linker length on domain swapping, finding that sufficiently long linkers can permit misfolded species to form in cases where they did not for the native spacing. Finally, we have developed a simple model for predicting the stability of misfolded intermediates (circular permutants of native), which should prove useful for determining whether a given protein may be susceptible to this type of misfolding.

## Supporting Information

S1 Text Thermodynamic and kinetic properties of all the systems. The melting temperature of single domain folding from umbrella sampling simulations (Table A). The mean first passage time of the folding simulation of the central domains (Table B). Free energy profile of WT (single domain) and its circular permutants (Fig A). The spread of native contacts formed at a given Q (Fig B). Free energy profile of the circular permutant of Ubiquitin with different linker lengths (Fig C). Free energy profile of the circular permutant of TNfn3 withdifferent linker lengths (Fig D).
(PDF)
