# Supplementary material for: Structural Determinants of Misfolding in Multidomain Proteins
Source: PLoS Comput Biol. 2016 May 10;12(5):e1004933. doi: 10.1371/journal.pcbi.1004933 (PMC4862688; doi:10.1371/journal.pcbi.1004933)
Supplement: S1 Text — The melting temperature of single domain folding from umbrella sampling simulations (Table A). The mean first passage time of the folding simulation of the central domains (Table B). Free energy profile of WT (single domain) and its circular permutants (Fig A). The spread of native contacts formed at a given Q (Fig B). Free energy profile of the circular permutant of Ubiquitin with different linker lengths (Fig C). Free energy profile of the circular permutant of TNfn3 withdifferent linker lengths (Fig D). (PDF) [file pcbi.1004933.s001.pdf]

# Supporting information for: “Structural determinants of misfolding in multidomain proteins”

Pengfei Tian[1], and Robert B. Best[1,*]

**1 Laboratory of Chemical Physics, National Institute of Diabetes and Digestive and Kidney Diseases, National Institutes of Health, Bethesda, MD 20892.**
*** robertbe@helix.nih.gov**

## Supporting Tables

**Table A. Melting temperature of single domain folding from umbrella sampling simulations.**

| Protein | Melting temperature (K) |
|---|---|
| SH3 | 327.0 |
| PDZ | 310.0 |
| TNfn3 | 297.0 |
| UBQ | 306.0 |
| SH2 | 312.0 |
| Titin I27 | 310.0 |
| GB1 | 320.0 |

**Table B. The mean first passage time ($\tau_{\mathbf{mfpt}}$) of the folding simulation of the central domains.** $k$ is the position of the cut of the circular permutation; $\tau_{\text{mfpt}}$ is estimated by the maximum likelihood method [1] based on 128 parallel simulations (12 microsecond for each). $\tau_{\text{mfpt}} = [N_{\text{fold}}\tau_{\text{fold}} + (N - N_{\text{fold}})t_{\text{sim}}]/N_{\text{fold}}$, where N is the total number of trajectories (N=128), $N_{\text{fold}}$ trajectories fold with an average folding time $\tau_{\text{fold}}$, $t_{\text{sim}}$ is the length of the simulation. The folding rate $k_{\text{f}}$ can be obtained by $1/\tau_{\text{mfpt}}$. Numbers in brackets show the correlation of that column with the first-passage time, for each protein.

| Protein | $k$ | $\tau_{\text{mfpt}}(ms)$ | $\Delta G_f$ (kcal/mol) | Population (%) |
|---|---|---|---|---|
| SH3 | 0 | 0.8 | 2.7 | 95.7 |
| | 18 | 25.4 | 3.4 | 1.1 |
| | 37 | 13. | 4.1 | 2.2 |
| | 46 | 12. | 3.0 | 1.1 |
| | | (1.0) | (0.80) | (-0.63) |
| PDZ | 0 | 1.1 | 2.5 | 88.7 |
| | 10 | 0.9 | 2.5 | 6.7 |
| | 23 | 5.8 | 3.6 | 2.3 |
| | 43 | 2.5 | 2.8 | 1.7 |
| | 60 | 15.3 | 4.2 | 0.7 |
| | 74 | 24.8 | 3.7 | 0.0 |
| | | (1.0) | (0.93) | (-0.88) |
| TNfn3 | 0 | 2.0 | 2.4 | 89.2 |
| | 16 | 1.5 | 2.4 | 0.0 |
| | 28 | 7.8 | 3.4 | 0.9 |
| | 43 | 13.9 | 3.9 | 2.3 |
| | 54 | 8.9 | 3.7 | 0.6 |
| | 66 | 10.7 | 3.5 | 1.3 |
| | 79 | 1.6 | 2.5 | 5.7 |
| | | (1.0) | (0.90) | (0.10) |
| SH2 | 0 | 1.2 | 2.6 | 91.7 |
| | 11 | 1.5 | 3.1 | 0.4 |
| | 24 | 1.0 | 3.2 | 0.0 |
| | 37 | 6.8 | 2.7 | 0.9 |
| | 49 | 2.9 | 3.3 | 1.1 |
| | 61 | 3.3 | 3.8 | 2.8 |
| | 72 | 4.4 | 3.9 | 2.6 |
| | 89 | 3.8 | 3.2 | 0.4 |
| | | (1.0) | (0.28) | (0.16) |
| Titin I27 | 0 | 0.6 | 2.5 | 92.0 |
| | 16 | 2.2 | 3.0 | 0.3 |
| | 28 | 1.6 | 2.8 | 3.1 |
| | 37 | 1.8 | 2.8 | 2.9 |
| | 53 | 2.1 | 3.0 | 0.2 |
| | 64 | 7.3 | 3.0 | 0.4 |
| | 76 | 4.0 | 2.8 | 2.0 |
| | | (1.0) | (0.69) | (-0.67) |

# Supporting Figures

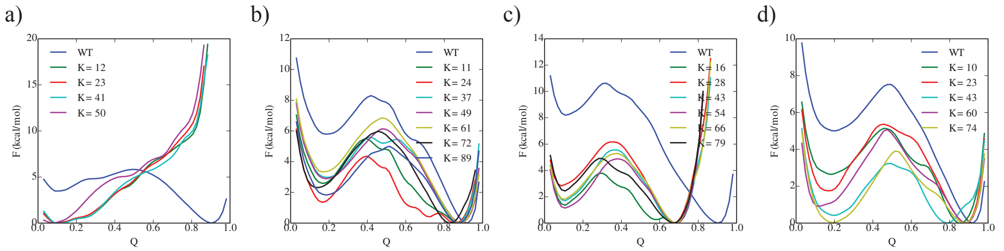


**Figure A. Free energy profile of WT (single domain) and its circular permutants.** a) GB1, b) SH2, c) TNfn3 and d) PDZ. The free energy is calculated at 305K, 294K, 275K, and 295K for GB1, SH2, TNfn3 and PDZ respectively.

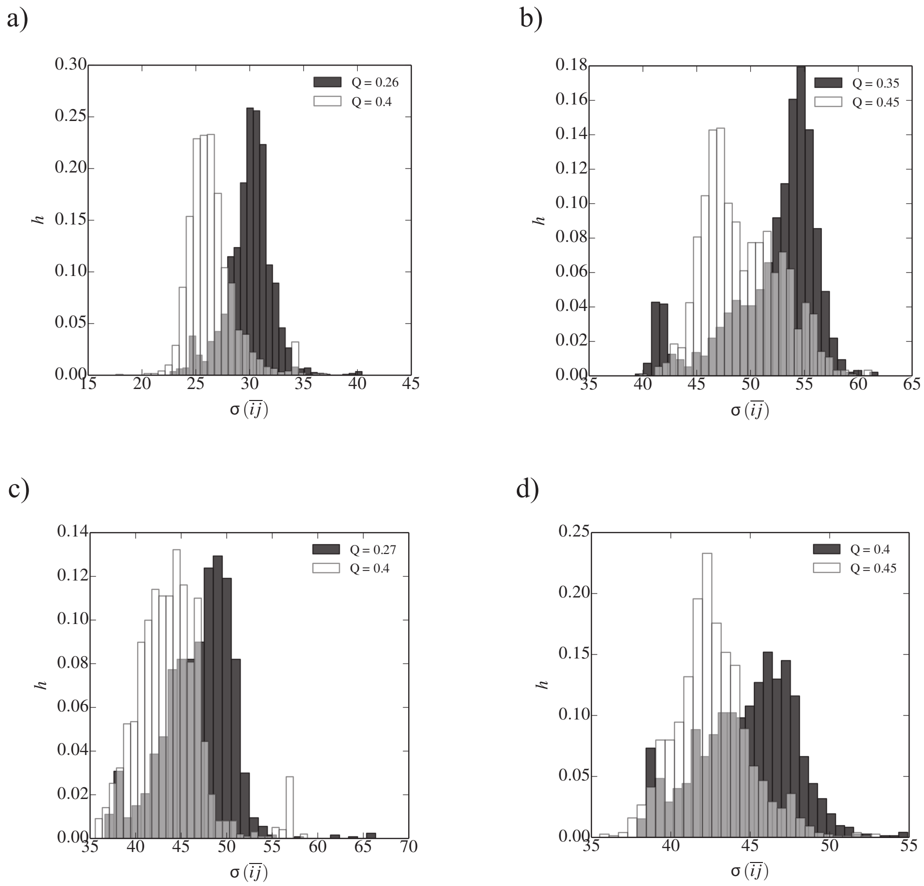


**Figure B. The spread of native contacts formed at a given $Q$.** We define the spread $\sigma(\bar{ij}) = [1/N \sum_{i,j}(i^2 + j^2)/2 - \bar{ij}^2]^{1/2}$ from the tandem dimer simulations (corresponding to the main text Fig. 8). The distributions of $\sigma(\bar{ij})$ of the SH3 (a), SH2 (b), TNfn3 (c) and PDZ (d) are extracted at two different Q (0.27 and 0.4) on the folding pathway.

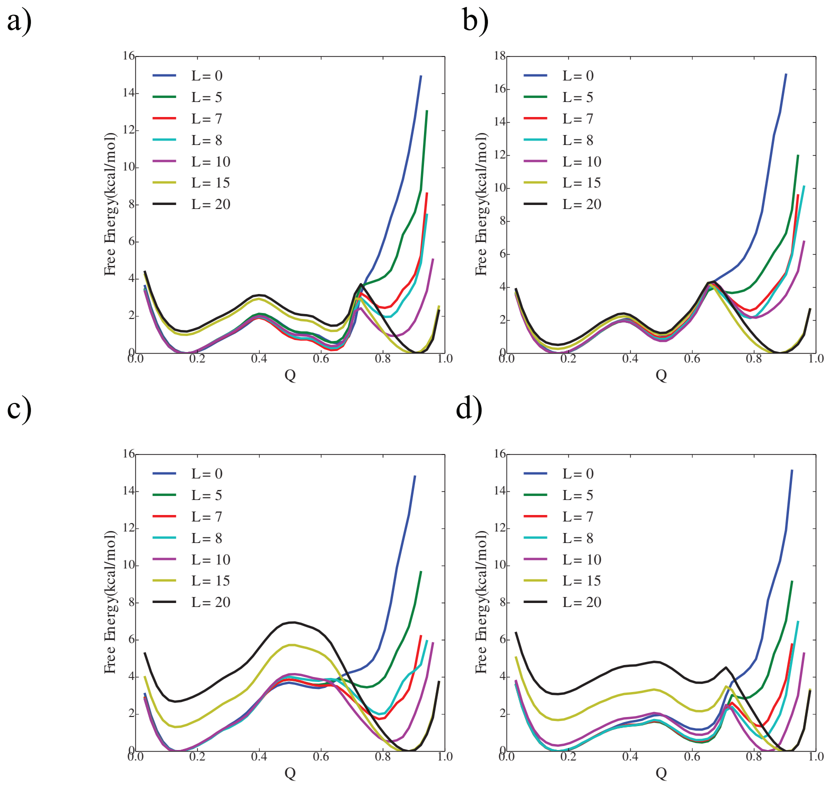


**Figure C. Free energy profile of the circular permutant of Ubiquitin with different linker lengths.** a) - d) corresponds to all the possible circular permutants of Ubiquitin indicated by the cut position K = 9, 21, 36 and 61 respectively.

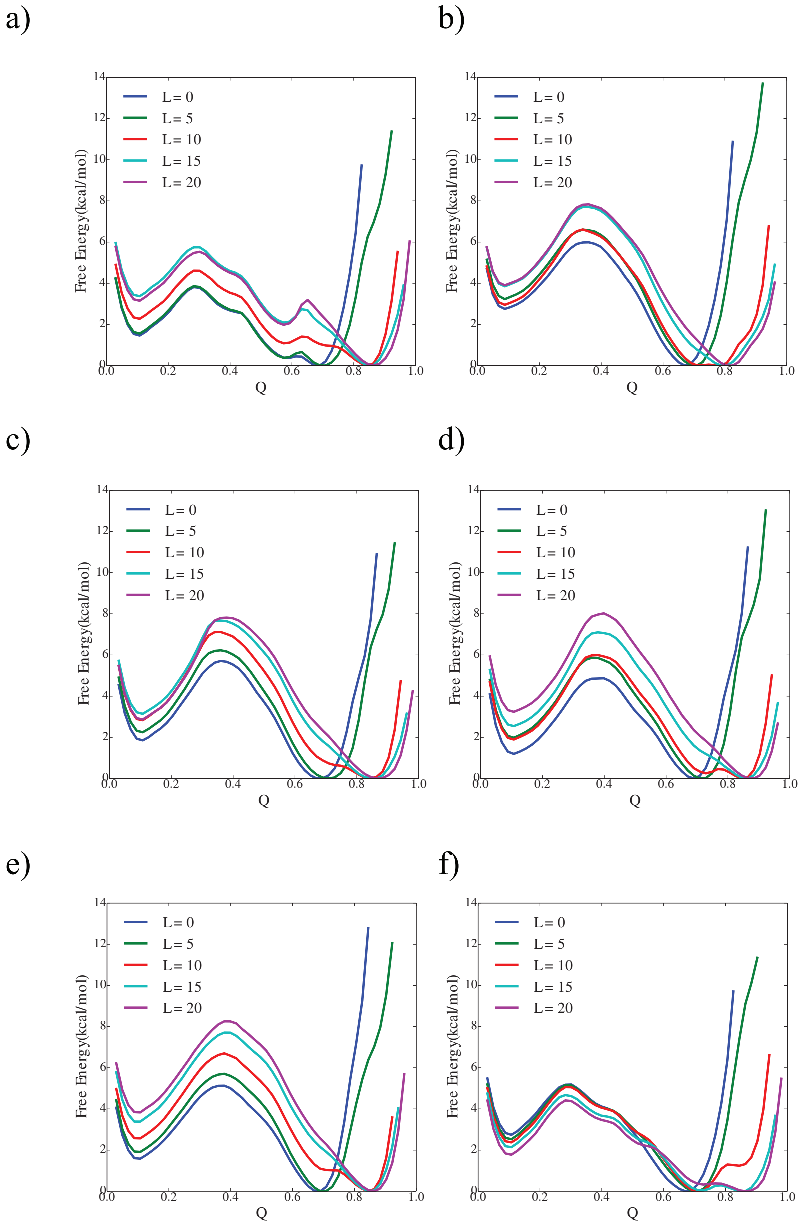


**Figure D. Free energy profile of the circular permutant of TNfn3 with different linker lengths.** a) - f) corresponds to all the possible circular permutants of TNfn3 indicated by the cut position K = 16, 28, 37, 53, 64 and 76 respectively.

## References

1. De Sancho D, Mittal J, Best RB. Folding kinetics and unfolded state dynamics of the GB1 hairpin from molecular simulation. J Chem Theor Comput. 2013;9(3):1743–1753.
